# Machine Learning of Band Gaps and Thermodynamic Stability in A_2_BB′X_6_ (X = F, Cl, Br, I) Double Perovskites with Uncertainty-Aware Multi-Objective Screening

**DOI:** 10.3390/nano16191217

**Published:** 2026-09-26

**Authors:** Qilin Wei, Haiyan Zhang, Chengyu Peng, Ye Tian, Yan Zhang, Yongmeng Yang, Menghong Liu, Nianxi Xu

**Affiliations:** 1 Department of Automotive and Transportation Engineering, Jining Polytechnic, Jining 272000, China; qlwei@sdu.edu.cn (Q.W.);; 2 Traffic Information Engineering Institute, Guangxi Transport Vocational and Technical College, Nanning 530004, China; 3 School of Semiconductors and Physics, North University of China, Taiyuan 030051, China

**Keywords:** double perovskites, energy above hull, thermodynamic stability, feature ablation, conformal prediction

## Abstract

A_2_BB′X_6_ double perovskites offer broad compositional flexibility, requiring screening strategies that jointly consider electronic properties and thermodynamic stability. We developed a leakage-controlled machine learning workflow integrating canonical-formula grouping, robustness tests, descriptor-cost analysis, and prediction uncertainty. The dataset contains 1975 Materials Project entries represented by 398 descriptors. Extra Trees predicted band gaps with a held-out MAE of 0.2031 eV and R^2^ of 0.9437, whereas histogram gradient boosting predicted energy above the hull with an MAE of 0.02122 eV atom^−1^ and R^2^ of 0.8601. The stability classifier achieved ROC-AUC, PR-AUC, and MCC values of 0.9603, 0.9632, and 0.8192, respectively. Removing 39 relaxed-structure descriptors increased hull-energy MAE by approximately 12.5%, supporting a two-stage workflow of composition-based triage followed by structure-informed refinement. Chemical-distribution-shift tests revealed substantial performance degradation and defined the applicability domain. Seventeen held-out compounds met the screening criteria, representing retrospective recovery rather than prospective discovery. Path-specific phonon calculations for one Tier-1 candidate and two representative Tier-2 fluorides showed no imaginary modes along L–Γ–X–W, but cannot establish full-zone dynamical stability. Their HSE06+SOC gaps of 2.148–2.919 eV exceeded the original 1.0–2.0 eV window, underscoring the need for multi-fidelity validation. Overall, the workflow provides a reproducible and well-defined strategy for prioritizing and validating double perovskites.

## 1. Introduction

Metal-halide perovskites have emerged as highly promising multifunctional semiconductor materials for photovoltaics, light-emitting devices, photodetectors, and related optoelectronic technologies, owing to their tunable band structures and the ability to fabricate thin films at relatively low temperatures [1,2,3,4,5]. However, concerns regarding the toxicity and environmental persistence of lead-containing components, coupled with the need for enhanced chemical and thermodynamic stability, have driven the search for lead-free or low-lead alternatives [6,7,8]. A_2_BB′X_6_-type double perovskites offer a vast platform for exploration; researchers gain significant design flexibility by ordering a pair of chemically distinct B and B′ cations to replace the two equivalent metal sites in the parent ABX_3_ lattice [9,10]. This flexibility—spanning A-site cations, B/B′ oxidation states, and halide anion combinations—enables precise control over orbital interactions, band-edge positions, lattice geometry, and phase stability [11,12]. However, this compositional versatility also results in an immense chemical space that cannot be exhaustively explored through experimental synthesis or high-precision first-principles calculations alone.

Optoelectronic material selection is application-dependent [13,14]. Here, the primary context is absorber pre-screening for single-junction photovoltaics, for which the most relevant priority band is 1.1–1.6 eV and the detailed-balance optimum is near 1.34 eV. We retain a broader 1.0–2.0 eV point-prediction window only as a permissive first-pass range because semilocal-DFT band gaps are method-dependent and because the upper part of this range can also be relevant to tandem top absorbers. Thermodynamic proximity to the convex hull is treated as a separate requirement. Previous high-throughput and ML studies have already predicted stability and band gaps or combined multiple filters for halide double perovskites [15,16,17]. Accordingly, this work does not claim the first ML screen of this family. Its narrower contribution is a validation architecture that combines formula-level leakage control, explicit chemical-distribution-shift tests, descriptor-availability analysis, and prediction-level uncertainty in one reproducible benchmark [16,18,19,20].

Reliable ML-assisted prioritization depends on how data are partitioned, interpreted, and used [21,22,23]. Canonical-formula grouping prevents polymorphs or site-order variants of the same normalized composition from crossing partitions, but it does not by itself establish generalization to chemically dissimilar compositions. We therefore distinguish interpolation to unseen formulas from stronger chemical out-of-distribution performance and report leave-one-halide-family-out and composition-cluster-holdout tests. We also distinguish predictive association from causality: descriptor ablation and permutation importance are used to test model reliance and physical consistency, not to infer governing mechanisms. Finally, descriptor availability matters. Thirty-nine descriptors are derived from relaxed structures, whereas 359 can be obtained without the same relaxed-geometry inputs; their incremental value is therefore interpreted within a two-stage screening workflow. Point predictions alone do not communicate the reliability of decisions near operational thresholds, including E_hull_ = 0.05 eV atom^−1^ [24,25,26]. We use empirical split-conformal intervals and prediction sets to flag boundary-sensitive cases. Because the binary stability label is defined directly from E_hull_ ≤ 0.05 eV atom^−1^, predicted E_hull_ is the primary thermodynamic quantity and *p*(stable) is used as an auxiliary cross-task consistency and confidence filter, not as an independent thermodynamic observable or calibrated probability of physical stability [27,28]. The operational *p*(stable) cutoff of 0.70 is stricter than the conventional 0.50 classification boundary but is not a physical stability threshold; its sensitivity over 0.50–0.90 is reported in Table S23. The previous weighted composite score, which double-counted thermodynamic stability through both E_hull_ and *p*(stable), has been removed [29,30]. Candidates are now reported by transparent point criteria and an uncertainty tier rather than by arbitrary scalar weights [31,32].

This study therefore evaluates three questions: (i) how accurately descriptor-based models recover band gaps and stability-related labels under a formula-disjoint split; (ii) how performance changes when structural descriptors, sparse descriptors, or entire chemical families are withheld; and (iii) whether empirical uncertainty intervals and prediction sets alter the interpretation of held-out candidate prioritization. The 17 retained test compounds are used only as a retrospective case study. They are not presented as previously unexplored discoveries, and independent high-level calculations or experiments remain necessary before any materials claim can be made.

## 2. Results and Discussion

The workflow is summarized in Figure 1. A_2_BB′X_6_ structures and reported target values were obtained from the Materials Project [33,34]. The energy-above-hull target is the database-reported value constructed from the Materials Project phase-diagram and energy-compatibility framework; no custom hull restricted to a hand-selected set of decomposition products was used. Canonical formulas were grouped before splitting, all preprocessing operations were fitted only on the relevant training subset, and the fixed test set was opened only for final evaluation. Formula-grouped cross-validation, stronger chemical holdouts, missingness sensitivity, and empirical uncertainty analysis were then used to delineate the scope of the reported performance. The queried target fields were the Materials Project database-reported band gap and energy above hull values associated with the retained MP identifiers. No local reconstruction of a restricted competing-phase hull was used; the target completeness is therefore limited to the database snapshot represented by the curated dataset.

The task-specific partitions and target distributions are shown in Figure 2. The training, validation, and test sets follow an approximate 70%/15%/15% ratio, with no canonical formula shared across subsets. Band gaps range from 0.0002 to 8.3194 eV (median 2.4711 eV). E_hull_ is strongly right-skewed, ranges from 0 to 0.8171 eV atom^−1^, and has a median of 0.06176 eV atom^−1^. Stable-class fractions are 44.94%, 42.42%, and 43.99% in the training, validation, and test sets. These similar marginal distributions support comparison across the fixed partitions, but they should not be interpreted as evidence of chemical out-of-distribution generalization. The latter is examined separately below.

Comparative results on the validation set demonstrate the clear advantage of non-linear ensemble models (Figure 3a–c). For the band gap regression task, the mean absolute error (MAE) was 1.384 eV for the mean baseline model, 0.488 eV for Ridge regression, 0.281 eV for histogram gradient boosting, and 0.188 eV for Extra Trees. Consequently, the selected Extra Trees model reduced the validation-set MAE by 86% compared to the mean baseline and by 61% compared to Ridge regression. Regarding the “energy-above-hull” metric, the validation-set MAE decreased from 0.0625 eV/atom (mean baseline) to 0.0267 eV/atom (Ridge regression), 0.0218 eV/atom (Extra Trees), and 0.01844 eV/atom (histogram gradient boosting). In the stability classification task, the validation-set ROC-AUC value improved from 0.500 (majority baseline) to 0.916 (logistic regression), 0.940 (Extra Trees), and 0.947 (histogram gradient boosting).

A post hoc benchmark on the unchanged validation split adds random forests and kernel support-vector models (Table S11). Extra Trees remains best for band gap (MAE 0.1867 eV versus 0.2086 eV for RBF-SVR and 0.2526 eV for random forest). Histogram gradient boosting remains best for Ehull (MAE 0.01799 eV atom^−1^ versus 0.02012 for random forest and 0.03104 for RBF-SVR) and gives a stable ROC-AUC = 0.9492, comparable to RBF-SVC at 0.9483. Graph neural networks are not included in this like-for-like table because they require raw structures and a different representation/training protocol; comparison with them is identified as a separate future benchmark. A bounded post hoc parameter grid shows that the conclusions are stable across 200–800 trees and neighboring learning-rate/leaf-node settings (Table S12).

On the held-out test set, the Extra Trees band gap model achieved an MAE of 0.2031 eV, a root mean square error (RMSE) of 0.3915 eV, and an R^2^ value of 0.9437 (Figure 3d and Table 1). Although a few compounds with unique chemical properties yielded large residuals (Figure S1), the majority of data points clustered closely around the ideal parity line. Predictions from the histogram gradient boosting model for “energy above hull” yielded an MAE of 0.02122 eV/atom, an RMSE of 0.03550 eV/atom, and an R^2^ of 0.8601 (Figure 3e). The dispersion of residuals increased slightly with higher predicted hull energies, indicating the presence of heteroscedasticity in the high-energy tail (Figure S2). The robust classifier achieved an ROC-AUC of 0.9603 and a PR-AUC of 0.9632 (Figure 3f and Figure S3). At the threshold of 0.555 determined using the validation set, the confusion matrix for the test set showed 155 true negatives (TNs), 110 true positives (TPs), eight false positives (FPs), and 18 false negatives (FNs) (Figure S4). The metrics were as follows: precision 0.9322, recall 0.8594, F1 score 0.8943, balanced accuracy 0.9051, and MCC 0.8192. The high ROC-AUC and PR-AUC values indicate the model’s excellent probability ranking capability, while the MCC confirms its balanced performance across different thresholds.

The right-skewed E_hull_ target creates a trade-off between near-hull accuracy and the high-energy tail. In a matched post hoc sensitivity rerun, log1p transformation improves overall MAE from 0.02081 to 0.01973 eV atom^−1^ and near-hull MAE from 0.01695 to 0.01425 eV atom^−1^, but increases tail MAE (reference E_hull_ > 0.10 eV atom^−1^) from 0.02972 to 0.03238 eV atom^−1^. Yeo–Johnson transformation and threefold tail weighting do not improve the tail (Table S16). We therefore retain the untransformed model for continuity and report the transformation results as a sensitivity analysis rather than implying uniformly improved robustness.

Feature ablation shows property-specific differences in predictive reliance (Figure 4a–c). For band gap prediction, removing the 39 relaxed-structure descriptors changes the test MAE only from 0.2031 to 0.2044 eV and R^2^ from 0.9437 to 0.9422; structural descriptors alone yield MAE = 0.9474 eV. These results indicate that, within this fitted model and dataset, most of the recoverable band gap signal is carried by chemical and electronic descriptors.

Relaxed-structure information contributes more to the thermodynamic tasks. Removing the 39 structural descriptors increases the test E_hull_ MAE from 0.02122 to 0.02389 eV atom^−1^ (approximately 12.5%) and decreases R^2^ from 0.8601 to 0.8156. For stability classification, their removal changes ROC-AUC from 0.9603 to 0.9522 and MCC from 0.8192 to 0.7786. These descriptors include relaxed lattice parameters, bond-length summaries, and geometry measures and therefore are not available for a genuinely unrelaxed hypothetical composition without a structural model or a relaxation calculation. We consequently frame the 359-feature non-structural model as a lower-cost first-stage model and the full 398-feature model as second-stage refinement; we do not claim that the full workflow eliminates the need for DFT relaxation.

Median imputation was selected because it is robust to skew and can be fitted entirely within each training fold; missingness indicators retain information about whether a value was absent. The two descriptors with more than 20% missingness were also removed in a sensitivity analysis. Across the fixed test sets, the resulting changes are small: band gap MAE changes from 0.2013 to 0.2030 eV, E_hull_ MAE from 0.02101 to 0.02123 eV atom^−1^, and stability ROC-AUC from 0.9607 to 0.9604. Removing all eight descriptors with any training missingness gives similarly small changes (Table S10), indicating that the main conclusions do not depend on median-filled sparse variables.

Individual permutation importance identifies descriptors on which the fitted predictions depend. For band gaps, high-ranked variables include B–X electronegativity differences, B-site d-electron statistics, valence-electron counts, and Mendeleev number summaries. These associations are physically consistent with established orbital and bonding arguments, but they do not establish that the descriptors causally govern the band gap.

For E_hull_ and stability classification, relaxed A–X geometry, cation-mass ratios, B-site ionization energies, oxidation-state summaries, and radius matching are prominent. To reduce instability caused by correlated predictors, we additionally permuted descriptor families jointly. On the validation set, joint permutation of elemental/exchange-invariant descriptors increased band gap and E_hull_ MAE by 1.268 and 0.0337, respectively, and decreased stability ROC-AUC by 0.174; joint permutation of the structural group increased E_hull_ MAE by 0.0243 and decreased stability ROC-AUC by 0.118 (Table S17). These grouped values quantify predictive dependence at the family level. They are not causal effect estimates, and the interpretation is limited to physical consistency with established knowledge [35,36,37].

Formula-grouped repeated cross-validation gives band gap MAE = 0.2113 ± 0.0179 eV, E_hull_ MAE = 0.02172 ± 0.00231 eV atom^−1^, and stability ROC-AUC = 0.9576 ± 0.0088 across 12 folds (Figure 5a–c, Table 1). Stronger distribution-shift tests are markedly harder. When one halide family is excluded from training, band gap MAE ranges from 0.274 eV (Br) to 1.086 eV (F), E_hull_ MAE from 0.0152 eV atom^−1^ (Br) to 0.0590 eV atom^−1^ (F), and stability ROC-AUC from 0.855 (F) to 0.946 (Br). Five composition-cluster holdouts show still larger and heterogeneous degradation (Table S14). These results demonstrate that exact-formula grouping controls a specific leakage mode but does not guarantee chemical extrapolation. Halide-resolved fixed-test results are also reported in Table S13; the lowest subgroup band gap R^2^ occurs for iodides (0.726, *n* = 30).

Bootstrap intervals quantify finite-test-set uncertainty. The 95% intervals are 0.1633–0.2468 eV for band gap MAE, 0.01835–0.02429 eV atom^−1^ for E_hull_ MAE, and 0.9362–0.9794 for stability ROC-AUC. Empirical 90% conformal coverages are 88.34% (197/223) for band gap, 87.97% (256/291) for E_hull_, and 93.13% (271/291) for stability, with the band gap prediction intervals shown in Figure 5d. Exact 95% binomial intervals are 83.38–92.24%, 83.67–91.48%, and 89.58–95.75%, respectively; all include 90%, and two-sided tests against 0.90 give *p* = 0.433, 0.241, and 0.078 (Table S18). Thus, the observed shortfalls for the two regression tasks are not statistically distinguishable from 90% at the 5% level. Nevertheless, the guarantees are marginal and require exchangeability; they need not hold within rare families or under chemical shift. Because the same validation partition contributed to model comparison before empirical calibration, we treat these intervals as uncertainty summaries rather than claiming an untouched-calibration finite-sample guarantee.

The held-out labeled set is used as a retrospective prioritization case study (Figure 5e,f). Seventeen compounds satisfy the point criteria *p*(stable) ≥ 0.70, predicted E_hull_ ≤ 0.05 eV atom^−1^, and predicted band gap of 1.0–2.0 eV; 14 also satisfy all corresponding Materials Project reference criteria (82.4%). No composite score is used. Instead, predicted E_hull_ is the primary thermodynamic quantity, *p*(stable) is an auxiliary cross-task consistency and confidence filter, and uncertainty creates two tiers. The 0.70 cutoff is an operational confidence filter rather than a physical boundary; varying it from 0.50 to 0.90 changes the broader retained list from 19 to 14 compounds, while the four Tier-1 candidates remain unchanged because all have *p*(stable) > 0.99 (Table S23). Four compounds—Rb_2_NaRhF_6_, Rb_2_InAgCl_6_, Cs_2_KCeBr_6_, and Cs_2_NaTlCl_6_—also have a 90% upper E_hull_ bound below 0.05 eV atom^−1^ and a singleton stable prediction set (Tier 1). The other 13 meet the point criteria but have an interval crossing the E_hull_ threshold (Tier 2). Compounds with predicted gaps of 1.1–1.6 eV are marked as the primary single-junction photovoltaic priority subset. Because reference labels already exist, neither tier constitutes prospective discovery [38,39].

We next performed independent first-principles calculations for three representative fluoride candidates selected to span both uncertainty tiers and the chemically challenging fluoride family: Rb_2_NaRhF_6_ (Tier 1), Cs_2_NaRhF_6_ (Tier 2), and Rb_2_CoAgF_6_ (Tier 2). Their PBE gaps are 1.75, 1.71, and 1.18 eV, respectively. HSE06 increases the gaps to 2.92, 2.85, and 2.15 eV, while inclusion of SOC changes them only slightly to 2.919, 2.849, and 2.148 eV (Figure 6). Thus, the newly calculated high-level gaps all lie above the 1.0–2.0 eV database-level screening window. This outcome does not invalidate the retrospective benchmark, whose target is the Materials Project band gap; instead, it directly demonstrates the functional dependence of the calculated band gaps and shows why shortlisted compounds require multi-fidelity re-evaluation before application-specific claims.

Harmonic phonon dispersions for the same three compounds show no modes below 0 THz along the sampled L–Γ–X–W path (Figure 7). The acoustic branches approach zero at Γ, as expected, without a finite imaginary mode on this path. Because only the displayed high-symmetry path was examined, instabilities at unsampled wavevectors elsewhere in the Brillouin zone cannot be excluded. These calculations therefore establish only the absence of imaginary modes along the sampled path.

The target values inherit the methodological approximations and heterogeneities of the underlying electronic-structure calculations [40,41]. The Cs_2_RbCeCl_6_ outlier (predicted 1.235 eV versus a Materials Project reference of 4.120 eV) contains Ce, for which localized 4f states, the treatment of on-site correlation, magnetic ordering, spin–orbit coupling, and functional-dependent band ordering can produce behavior not represented by global descriptors. Because the reference entry is a pristine bulk calculation, we do not invoke defect configurations as the primary explanation. E_hull_ = 0.05 eV atom^−1^ remains an operational screen rather than a synthesis criterion: finite-temperature free energies, kinetics, chemical potentials, disorder, and synthesis pathways are not captured. The database-reported hull uses the available Materials Project phase-diagram entries and compatibility corrections, but its completeness is limited by the database snapshot [42,43]. The added halide and cluster holdouts also show that uncertainty and accuracy deteriorate under chemical shift [44].

The 17-compound candidate evaluation remains retrospective against existing computational labels, but the added calculations provide an independent compound-specific check for three representative fluorides. The result is deliberately interpreted in two parts: no imaginary modes were observed along the sampled L–Γ–X–W path, whereas the HSE06+SOC gaps move above the database-level screening window and therefore prevent an unqualified photovoltaic-candidate claim. Only Rb_2_NaRhF_6_ among the four Tier-1 compounds has been examined in this follow-up set; Cs_2_NaRhF_6_ and Rb_2_CoAgF_6_ are representative Tier-2 shortlisted fluorides. The remaining Tier-1 compounds—Rb_2_InAgCl_6_, Cs_2_KCeBr_6_, and Cs_2_NaTlCl_6_—and other chemical families still require independent high-level assessment.

## 3. Conclusions

This study establishes a leakage-controlled benchmark for predicting band gaps and stability-related labels of A_2_BB′X_6_ double perovskites. Canonical-formula grouping prevents a specific polymorph/composition leakage mode, while leave-one-halide and composition-cluster tests show that performance under chemical shift is substantially weaker. Ablation supports a two-stage workflow: composition-derived descriptors provide broad triage, and relaxed-structure descriptors improve second-stage thermodynamic estimates. Individual and grouped permutation analyses show predictive associations consistent with established chemical knowledge but do not establish causal mechanisms. Empirical uncertainty intervals convert the held-out case study into an explicit two-tier prioritization and eliminate the earlier redundant weighted score. Independent calculations for one Tier-1 candidate and two representative Tier-2 shortlisted fluorides show no imaginary modes along the sampled path. Their HSE06+SOC gaps are 2.148–2.919 eV, above the original database-level window. The combined evidence therefore supports the workflow as a prioritization and validation framework while showing that complete vibrational assessment and high-level electronic calculations remain essential before application-specific materials claims are made.

## Figures and Tables

**Figure 1:**
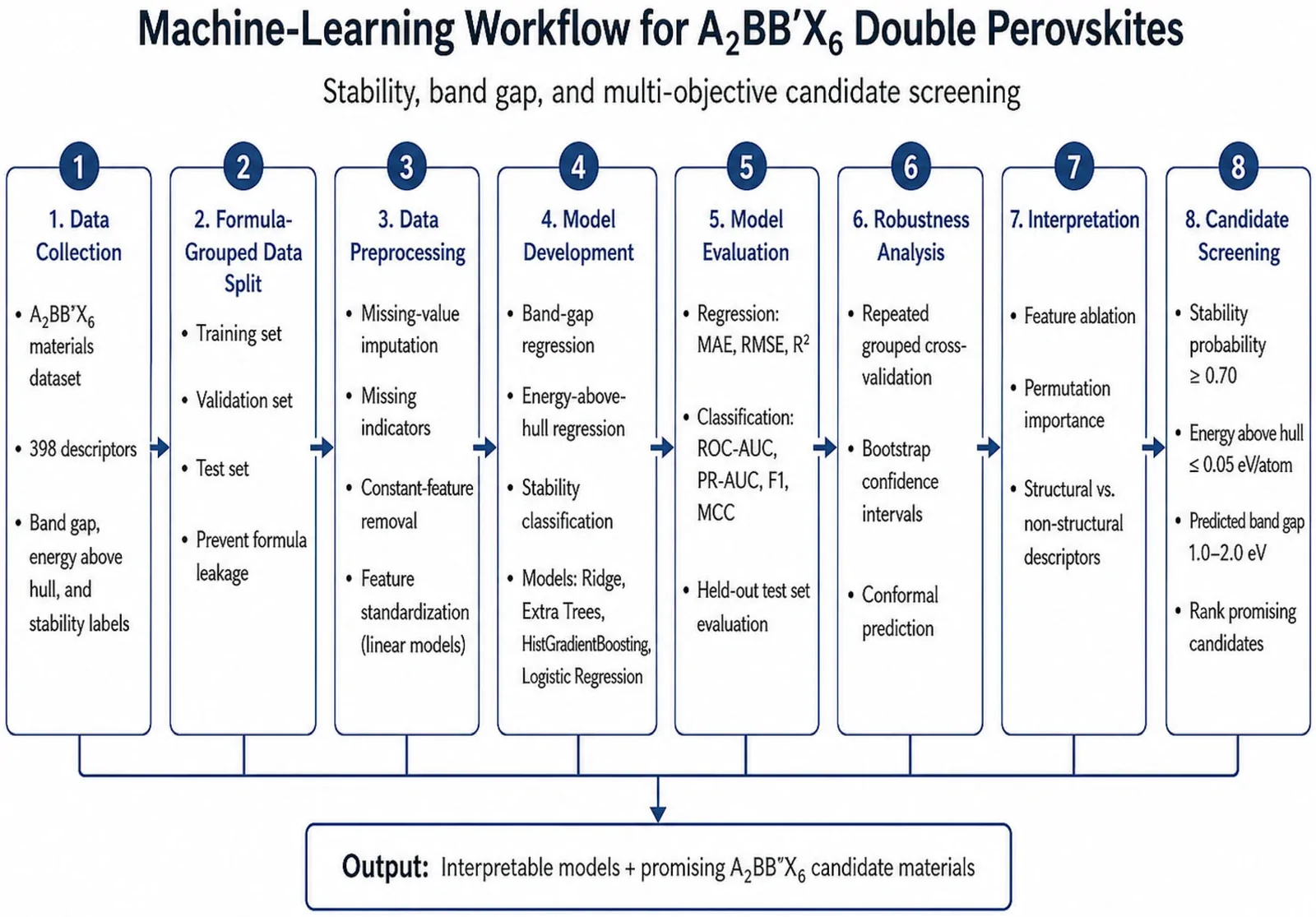
Machine learning workflow for property prediction and retrospective held-out prioritization of A_2_BB′X_6_ double perovskites. The workflow integrates dataset construction, canonical-formula grouping, leakage-controlled preprocessing, model development, held-out evaluation, chemical-distribution-shift tests, descriptor-cost analysis, grouped interpretation, and uncertainty quantification.

**Figure 2:**
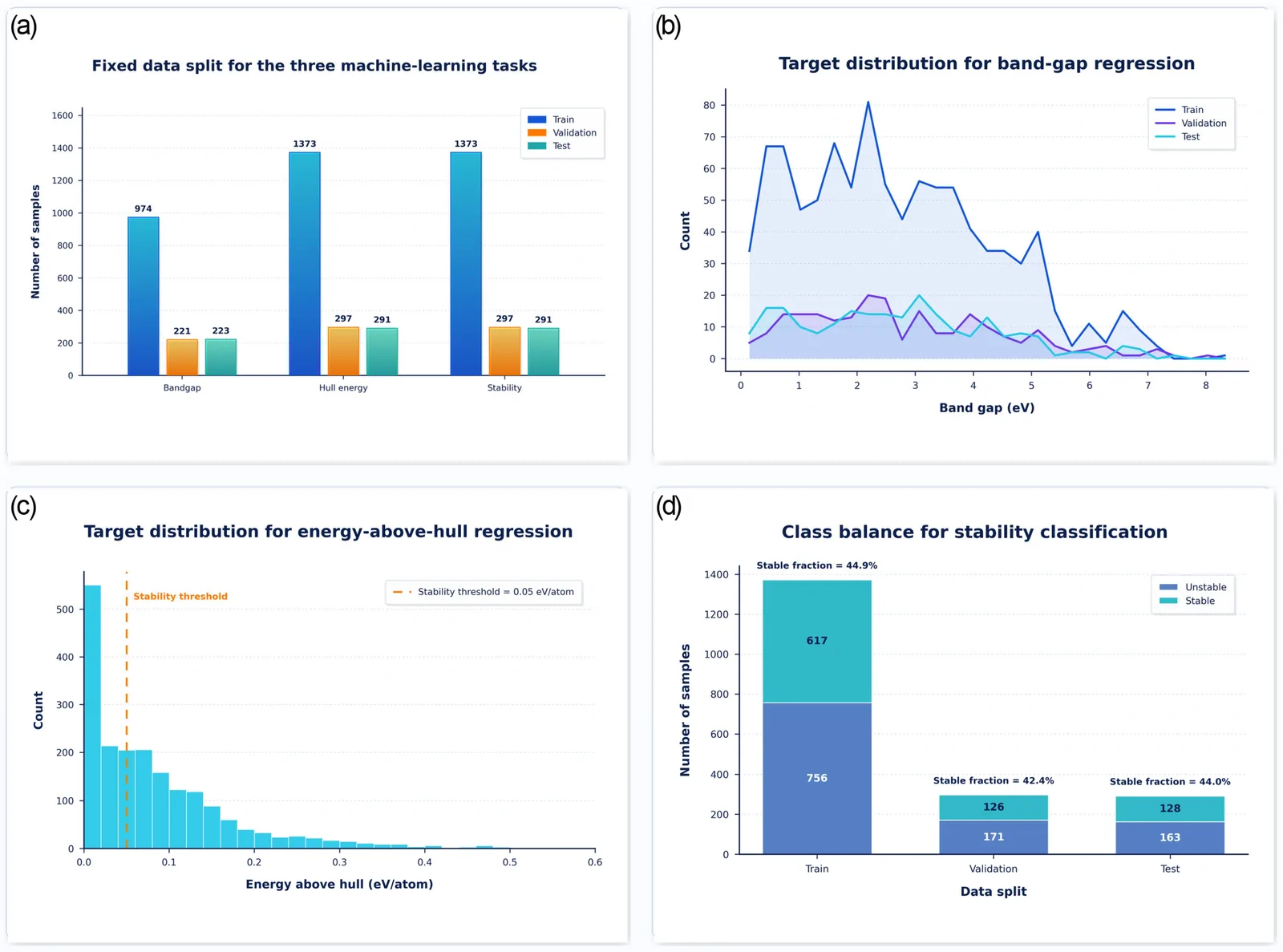
Dataset partitioning and target-property distributions for the three machine learning tasks. (**a**) Numbers of samples assigned to the training, validation, and held-out test sets for band gap regression, energy-above-hull regression, and stability classification. (**b**) Distribution of band gap values across the fixed subsets. (**c**) Distribution of energy above hull, with the 0.05 eV atom^−1^ stability threshold indicated. (**d**) Stable and unstable class counts in the three subsets. Canonical-formula grouping prevents composition overlap across the partitions.

**Figure 3:**
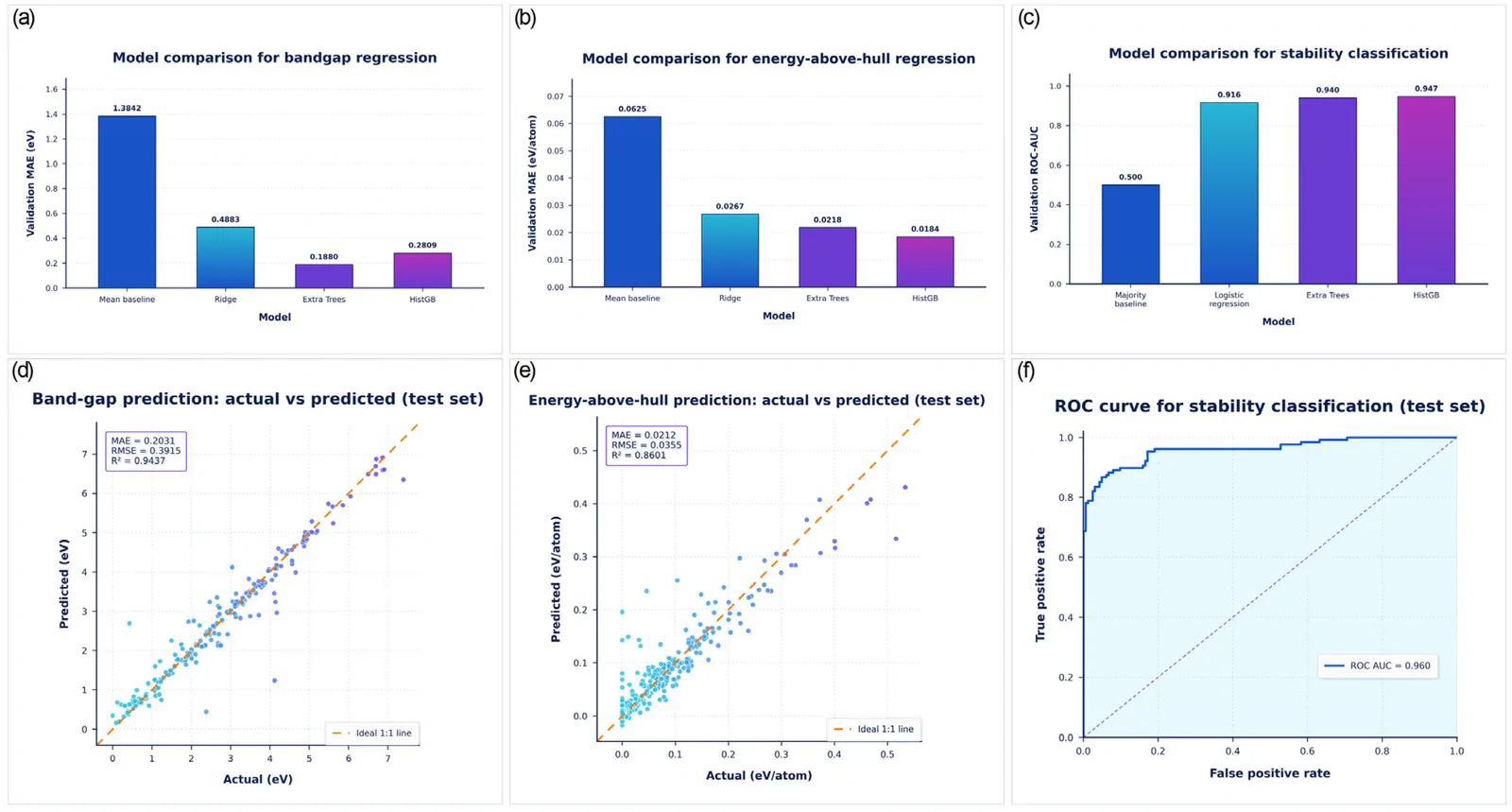
Model selection and predictive performance for band gap regression, energy-above-hull regression, and stability classification. (**a**–**c**) Validation-set comparison of candidate algorithms. (**d**) Predicted versus calculated band gaps on the held-out test set. (**e**) Predicted versus calculated energies above hull on the held-out test set. The dot colors encode the calculated values, with cyan indicating lower values and violet indicating higher values. (**f**) Receiver operating characteristic curve for held-out stability classification. All model choices and the classification threshold were fixed before test-set evaluation.

**Figure 4:**
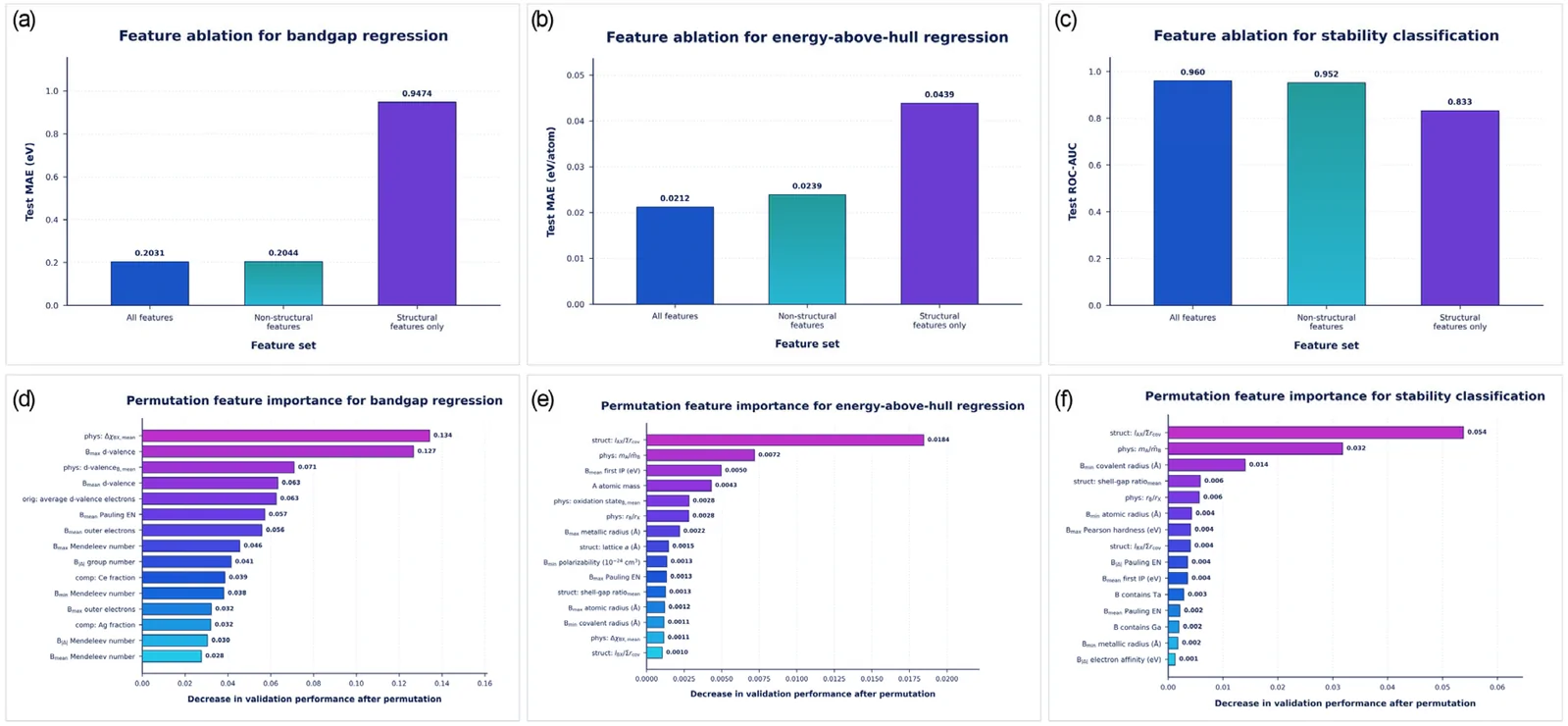
Feature ablation and permutation-importance analysis for the three prediction tasks. (**a**–**c**) Test performance using all descriptors, non-structural descriptors, or structural descriptors only for band gap regression, energy-above-hull regression, and stability classification, respectively. (**d**–**f**) Validation-set permutation importance for the selected models. Structural information provides complementary value for thermodynamic tasks, whereas band gap prediction relies predominantly on B/B′ electronic and electronegativity descriptors.

**Figure 5:**
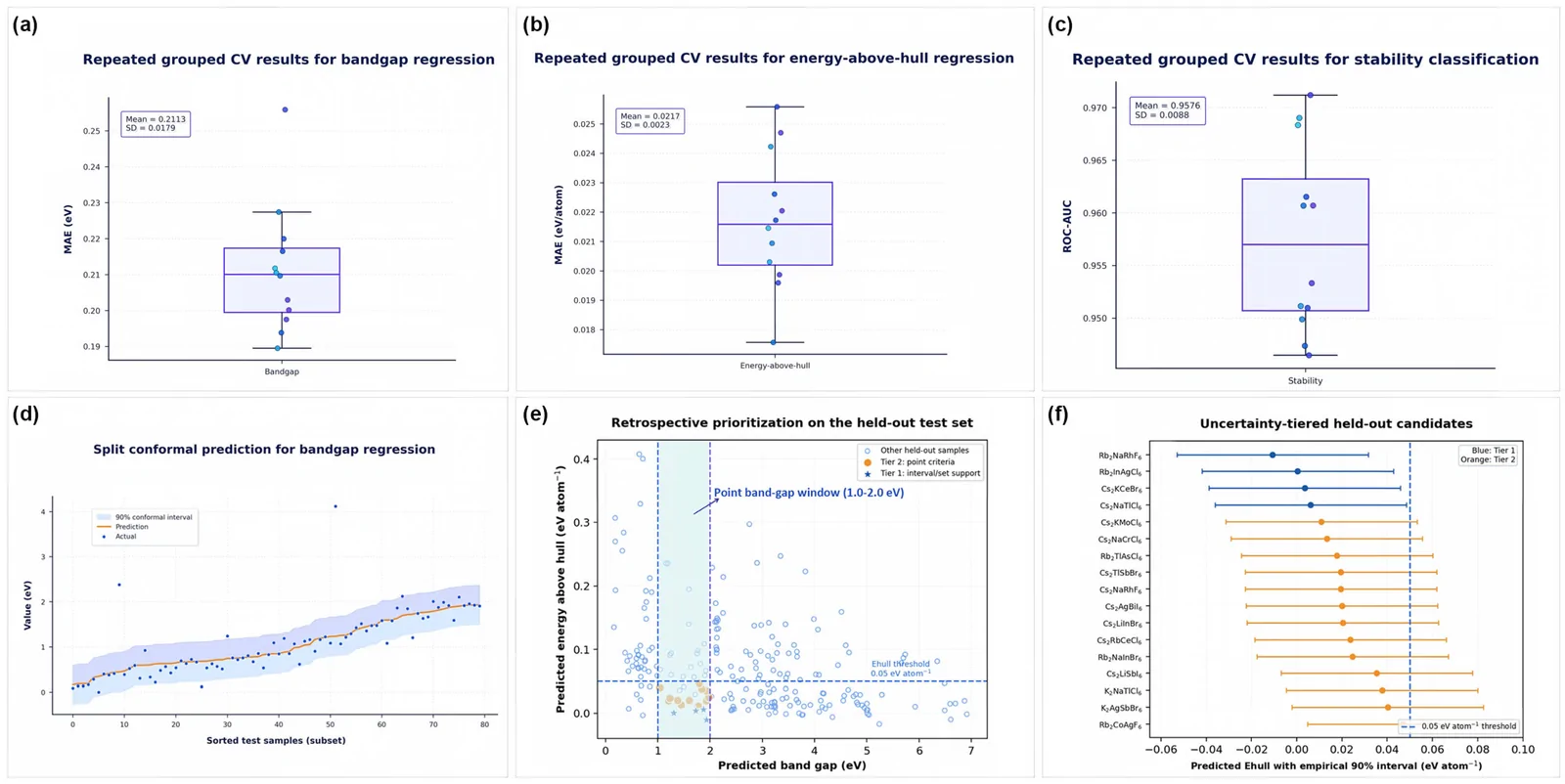
Robustness assessment and retrospective prioritization of held-out A_2_BB′X_6_ compounds. (**a**–**c**) Repeated formula-grouped cross-validation distributions for band gap MAE, E_hull_ MAE, and stability ROC-AUC.The colored circles represent metric values from individual repeated cross-validation runs. (**d**) Empirical split-conformal prediction for band gap regression; the blue-to-purple shaded band represents the empirical 90% conformal prediction interval. (**e**) Point-criterion selection in predicted band gap and E_hull_ space, distinguishing interval-supported Tier 1 from boundary-sensitive Tier 2. (**f**) Predicted E_hull_ values and empirical 90% intervals for the 17 retained compounds; no weighted composite score is used.

**Figure 6:**
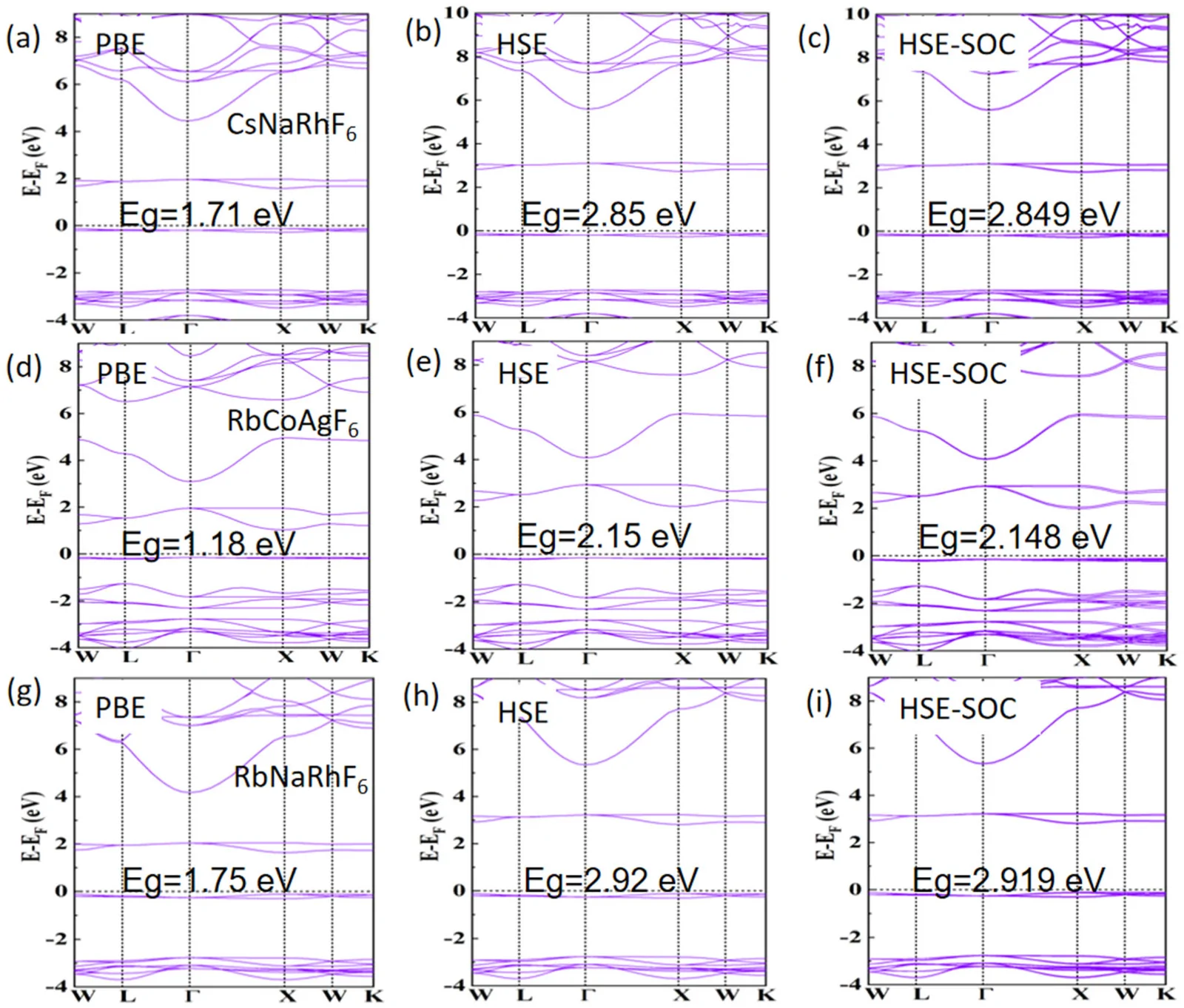
Independent electronic-structure validation for representative shortlisted fluorides. PBE, HSE06, and HSE06+SOC band structures are shown for (**a**–**c**) Cs_2_NaRhF_6_, (**d**–**f**) Rb_2_CoAgF_6_, and (**g**–**i**) Rb_2_NaRhF_6_. Energies are referenced to the Fermi level.

**Figure 7:**
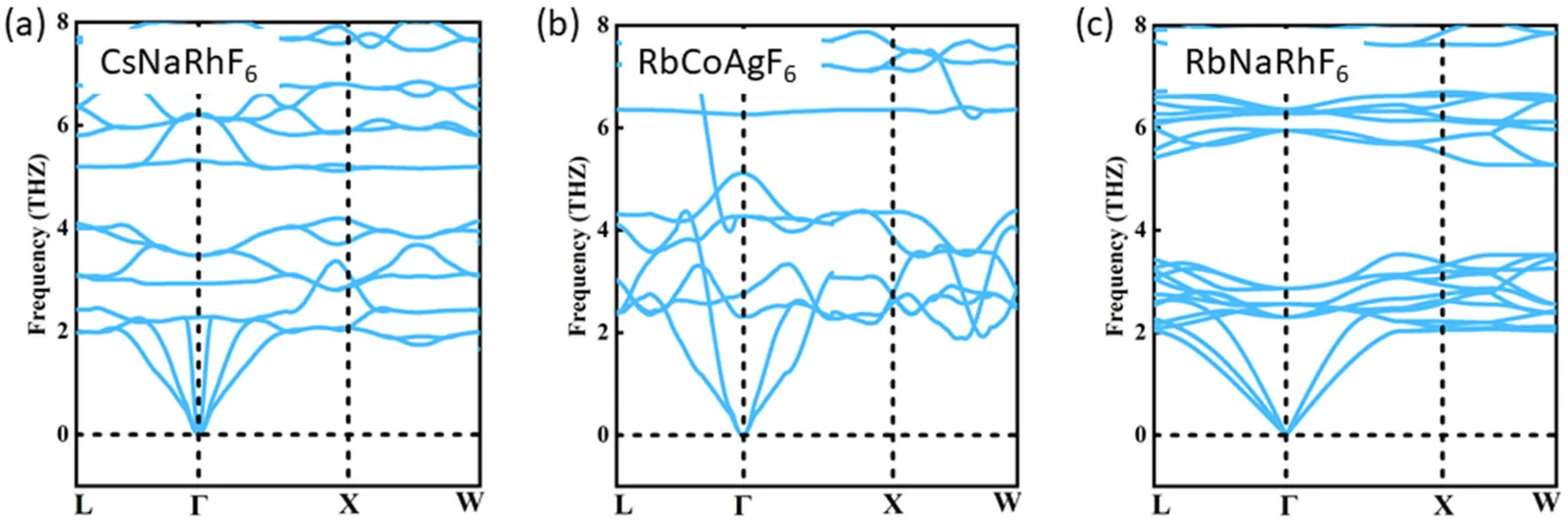
Harmonic phonon dispersions of (**a**) Cs_2_NaRhF_6_, (**b**) Rb_2_CoAgF_6_, and (**c**) Rb_2_NaRhF_6_. No imaginary phonon mode is observed along the sampled L–Γ–X–W path.

**Table 1:** Predictive performance, cross-validation stability, and bootstrap uncertainty of the selected machine learning models.

Bootstrap 95% CI	Repeated Grouped CV	Held-Out Test Performance	Selected Model	Task
MAE 0.1633–0.2468 eV	MAE 0.2113 ± 0.0179 eV	MAE 0.2031 eV; RMSE 0.3915 eV; R^2^ 0.9437	Extra Trees	Band gap regression
MAE 0.01835–0.02429 eV atom^−1^	MAE 0.02172 ± 0.00231 eV atom^−1^	MAE 0.02122 eV atom^−1^; RMSE 0.03550; R^2^ 0.8601	HistGradientBoosting	Energy-above-hull regression
ROC-AUC 0.9362–0.9794	ROC-AUC 0.9576 ± 0.0088	ROC-AUC 0.9603; PR-AUC 0.9632; F1 0.8943; MCC 0.8192	HistGradientBoosting	Stability classification

## Data Availability

The processed datasets and code supporting the findings of this study are openly available at https://github.com/qilinwei0/Machine-Learning-of-Band-Gaps-and-Thermodynamic-Stability-in-A2BB-X6-X-F-Cl-Br-I-Double-Perovskites (accessed on 22 September 2026). The archive contains the task-specific modeling tables, feature manifest, canonical-formula split assignments, frozen validation and test predictions, candidate-screening results, supplementary sensitivity and distribution-shift outputs, executable scripts, and software requirements. The underlying Materials Project records can be retrieved using the Materials Project identifiers included in the archived tables.

## Supplementary Materials

The following supporting information can be downloaded at https://www.mdpi.com/article/10.3390/nano16191217/s1, Figure S1: Residuals for held-out band-gap prediction. Residuals are broadly centered on zero, but a small number of high-error samples create a heavy tail; Figure S2: Residuals for held-out energy-above-hull prediction. Dispersion increases with predicted energy, indicating moderate heteroscedasticity outside the near-hull region; Figure S3: Precision-recall curve for held-out stability classification. The test PR-AUC is 0.9632, substantially exceeding the positive-class prevalence; Figure S4: Confusion matrix for stability classification at the validation-selected threshold of 0.555. The matrix contains 155 true negatives, 110 true positives, 8 false positives, and 18 false negatives; Figure S5: Bootstrap 95% confidence interval for held-out band-gap MAE. The point estimate is 0.2031 eV and the percentile interval is 0.1633–0.2468 eV; Figure S6: Bootstrap 95% confidence interval for held-out energy above hull MAE. The point estimate is 0.02122 eV atom^−1^ and the percentile interval is 0.01835–0.02429 eV atom^−1^; Figure S7: Bootstrap 95% confidence interval for held-out stability ROC-AUC. The point estimate is 0.9603 and the percentile interval is 0.9362–0.9794; Figure S8: Split-conformal 90% prediction intervals for held-out energy-above-hull regression. The empirical coverage is 87.97% and the average interval width is 0.08456 eV atom^−1^; Figure S9: Distribution of split-conformal prediction-set sizes for stability classification. A total of 273 samples receive singleton sets and 18 receive two-label sets; Table S1: Task-specific datasets, fixed canonical-formula splits, and data-quality statistics; Table S2: Full validation and held-out test comparison of all benchmark models; Table S3: Feature-ablation results for all, non-structural, and structural-only descriptor sets; Table S4: Ten highest validation-set permutation importances for each selected model; Table S5: Repeated formula-grouped cross-validation summary; Table S6: Bootstrap percentile confidence intervals for held-out test metrics; Table S7: Split-conformal calibration and held-out coverage summary; Table S8: Held-out samples satisfying the point criteria, reported without a weighted composite score. Reference labels are shown only for retrospective evaluation; Table S9: Descriptors containing missing values in each task-specific dataset; Table S10: Independent-refit sensitivity to sparse-descriptor removal on the fixed test set. Values are compared with the matching all-feature baseline from the same refit; the frozen prediction files remain authoritative for the principal fixed-test metrics; Table S11: Expanded algorithm benchmark on the unchanged validation split; Table S12: Bounded post hoc hyperparameter robustness on the validation split; Table S13: Fixed-test performance by halide family; Table S14: Stronger chemical-distribution-shift tests. Clusters were defined without target labels from the 359 non-structural descriptors; Table S15: Fixed-test performance by training frequency of the B/B’-X combination; Table S16: E_hull_ target-skew sensitivity on the fixed test set (matched post hoc rerun); Table S17: Joint descriptor-family permutation importance on the validation split (10 repeats). Positive values indicate MAE increase or ROC-AUC decrease; Table S18: Exact inference for empirical coverage relative to the nominal 90% level; Table S19: Uncertainty-tiered retrospective prioritization of the 17 held-out samples passing point criteria. The reference-hit column is used only for post hoc evaluation; Table S20: Independent first-principles electronic validation and path-restricted phonon results for one Tier-1 candidate and two representative Tier-2 shortlisted fluorides; Table S21: Pseudopotentials used in the electronic-structure and DFPT phonon calculations; Table S22: Dependence of collinear spin-polarized PBE solutions on the initial Rh or Co magnetization. nspin = 2 was used without a fixed-total-magnetization constraint; Table S23: Sensitivity of the retained candidate count to the *p*(stable) threshold while the predicted band-gap criterion (1.0–2.0 eV) and predicted E_hull_ criterion (≤0.05 eV atom^−1^) are held fixed. The four Tier-1 candidates remain unchanged across all thresholds because each has *p*(stable) > 0.99. References [45,46,47,48,49,50,51,52,53,54,55,56,57,58,59,60,61,62,63,64,65] are cited in the Supplementary Materials.
