# Supplementary material for: Machine Learning of Band Gaps and Thermodynamic Stability in A2BB′X6 (X = F, Cl, Br, I) Double Perovskites with Uncertainty-Aware Multi-Objective Screening
Source: Nanomaterials (Basel). 2026 Sep 26;16(19):1217. doi: 10.3390/nano16191217 (PMC13667066; doi:10.3390/nano16191217)
Supplement: Supplementary file 1 [file nanomaterials-16-01217-s001.zip › nanomaterials-4561873-supplementary.pdf]

Supporting Information

Machine Learning of Band Gaps and Thermodynamic Stability in $A_2BB'X_6$ (X=F,Cl,Br,I) Double Perovskites with Uncertainty-Aware Multi-Objective Screening

Qilin Wei[1], Haiyan Zhang[1],Chengyu Peng[2],Ye Tian[3], Yan Zhang[1], Yongmeng Yang[1], Menghong Liu[1] and Nianxi Xu[1,*]

1 Department of Automotive and Transportation Engineering, Jining Polytechnic, Jining 272000, China;

2 Traffic Information Engineering Institute, Guangxi Transport Vocational and Technical College, Nanning 530004, China;

3 School of Semiconductors and Physics, North University of China, Taiyuan 030051, China.

* Correspondence: xnxlzhy999@126.com

Materials and Methods

Dataset and prediction tasks

This analysis used a curated master dataset of 1,975 $A_2BB'X_6$ Materials Project entries and 398 model descriptors. The band-gap task contains 1,418 non-metallic entries; E_{hull} regression and stability classification each contain 1,961 entries. Stability is an operational label defined directly as $E_{hull} \leq 0.05$ eV atom^{-1} and is not an independent physical target. The E_{hull} values are the Materials Project database-reported energy_above_hull values derived through its database-wide phase-diagram and energy-compatibility framework; no custom hull restricted to selected binary or ternary products was constructed. Database completeness is therefore that of the retrieved Materials Project snapshot. Identifiers and quality-control fields were excluded from the input matrix. The 0.05 eV atom^{-1} cutoff is a pragmatic prioritization threshold, not a guarantee of synthesizability [1–5].

The queried targets were the database-reported band_gap and energy_above_hull fields linked to the retained Materials Project identifiers. The energy above hull was not recomputed locally from a restricted set of binary or ternary products; database completeness is therefore limited to the Materials Project snapshot represented in the curated master table.

Formula-grouped splitting and leakage control

Canonical formulas were used as groups so that polymorphs or alternative site arrangements of one normalized composition remain in the same subset. The band-gap split contains 974/221/223 training/validation/test samples; the thermodynamic splits contain 1,373/297/291. No canonical formula crosses subsets. The training set was used for preprocessing and fitting, the validation set for algorithm comparison and threshold selection, and the fixed test set for final evaluation. Because the validation subset contributed to model comparison before empirical conformal calibration, the reported intervals and sets are presented as empirical uncertainty summaries; we do not claim the strict finite-sample guarantee associated with a completely untouched calibration set. Repeated grouped cross-validation merges training and validation while keeping the fixed test set excluded[6–11].

Descriptor preprocessing

All 398 descriptors were parsed numerically. Missing values were imputed with medians fitted only on the current training fold, and missingness indicators were added. Constant features were removed within the same pipeline. Eight variables contain missing values; two oxidation-state-derived variables reach 44.99% missingness in the band-gap subset and 34.37% in the thermodynamic subsets. Median imputation was chosen for robustness to skew and because it can be applied without target information. Sensitivity tests remove the two variables above 20% missingness and, separately, all eight variables with any training missingness (Table S10). Scaling was fitted only for Ridge, logistic, and support-vector models; tree models used original scales.

Models, selection criteria, and evaluation metrics

The original benchmark compared mean/majority baselines, Ridge or logistic regression, Extra Trees, and histogram gradient boosting. The revision adds random forests and RBF support-vector regression/classification on the unchanged validation split (Table S11). The final reported configurations are ExtraTreesRegressor(n_estimators = 400, max_features = 0.7,

random_state = 42) for band gap and HistGradientBoostingRegressor/Classifier(max_iter = 300, learning_rate = 0.05, max_leaf_nodes = 15, l2_regularization = 1.0, random_state = 42) for the thermodynamic tasks. No automated global hyperparameter optimization was used in the original analysis. Model class was selected on validation performance, and these moderate-capacity settings were fixed before test evaluation. The revision therefore reports a bounded post-hoc robustness grid—200/400/800 trees with max_features = 0.5/0.7/1.0, and learning_rate = 0.03/0.05/0.10 with 15/31 leaf nodes—rather than retroactively describing an optimization that was not performed (Table S12). The classification threshold 0.555 maximized validation MCC. Regression metrics are MAE, RMSE, and R^2; classification metrics include ROC-AUC, PR-AUC, F1, balanced accuracy, and MCC[12–14].

Feature ablation and permutation importance

Thirty-nine descriptors with the feat_struct_prefix were classified as relaxed-structure descriptors and 359 as non-structural. Full, non-structural-only, and structural-only models quantify incremental and standalone predictive information. The structural variables include lattice and relaxed-geometry quantities and therefore require either a relaxed structure or a surrogate structural model. The intended practical use is two-stage: 359 composition/electronic descriptors for broad lower-cost triage, followed by the 398-feature model after structural relaxation for a smaller subset. Individual permutation importance was computed on validation data. In addition, related descriptor families were jointly permuted using a common row permutation, preserving within-family dependence while breaking their joint association with the target (Table S17). Both analyses quantify predictive reliance, not causality[15–16].

Robustness and uncertainty quantification

Three repeats of four-fold canonical-formula-grouped cross-validation were performed on the development data. Stronger robustness checks additionally leave out one entire halide family and hold out one of five clusters obtained from the 359 non-structural descriptors (Table S14). Halide-resolved fixed-test metrics and performance stratified by the training frequency of the B/B′–X combination are reported in Tables S13 and S15. The right-skewed Ehull target is examined with log1p and Yeo–Johnson transforms and threefold weighting for training samples above 0.10 eV atom^{-1} (Table S16). Bootstrap intervals use 500 resamples. Empirical split-conformal intervals/sets use absolute-residual or probability nonconformity scores. Exact Clopper–Pearson intervals and binomial tests quantify whether observed coverage differs detectably from 90% (Table S18). These uncertainty summaries are marginal and do not imply uniform conditional coverage under chemical shift[17–19].

Retrospective held-out prioritization. The candidate exercise is restricted to already labeled samples shared by the three fixed test sets and is therefore retrospective. Point criteria are P(stable) ≥ 0.70, predicted E_{hull} ≤ 0.05 eV atom^{-1}, and predicted band gap of 1.0–2.0 eV. The previous weighted composite score has been removed because stability is defined from E_{hull} and the score consequently double-counted thermodynamic stability. Predicted E_{hull} is the primary thermodynamic variable; P(stable) is an auxiliary cross-task consistency and

confidence filter, not an independent thermodynamic observable or a calibrated probability of physical stability. The operational 0.70 cutoff is stricter than the conventional 0.50 boundary and removes borderline classifier outputs without excessive pruning; it is not a physically meaningful stability boundary. Table S23 shows that thresholds from 0.50 to 0.90 change the broader retained list from 19 to 14 compounds, whereas the four Tier-1 candidates remain unchanged because all have P(stable) > 0.99. Tier 1 requires the point criteria, an upper empirical 90% E_{hull} bound ≤ 0.05 eV atom^{-1}, and a singleton stable prediction set. Tier 2 satisfies the point criteria but has an interval crossing the E_{hull} boundary. The 1.1–1.6 eV subset is separately flagged for the primary single-junction photovoltaic context. Table S19 reports all 17 retained samples and their existing reference labels for post-hoc evaluation; none is claimed as a prospective discovery [20].

Software and reproducibility. The principal fixed-test metrics were calculated from frozen prediction files generated during the original end-to-end workflow and are the authoritative values reported in the main text. The sparse-feature and related sensitivity tables were generated by independently refitting the complete pipeline in a later analysis environment with the same split assignments, test compounds, feature definitions, model hyperparameters, and random seed. Small numerical differences can arise from software-version-dependent tree construction, histogram binning, missing-value imputation, and floating-point operations. The independent refits used Python 3.12.14, NumPy 2.3.5, pandas 2.2.3, SciPy 1.17.0, scikit-learn 1.8.0, and Matplotlib 3.10.8, with random seed 42. The band-gap MAE difference is 0.00183 eV (approximately 0.9%), and the E_{hull} MAE difference is approximately 2.1×10^{-4} eV atom^{-1}(approximately 1.0%); neither changes the sensitivity conclusions. Feature-removal effects are therefore evaluated against the matching all-feature independent-refit baseline within each sensitivity run.

Computational Methods:

First-principles calculations were performed within density-functional theory using the plane-wave pseudopotential implementation in Quantum ESPRESSO 7.2 [21]. The primitive 10-atom cell containing one $A_2BB'X_6$ formula unit was used throughout. Exchange and correlation were described with the PBE generalized-gradient approximation for semilocal calculations and the screened HSE06 hybrid functional for hybrid calculations. Scalar-relativistic norm-conserving PBE pseudopotentials were used for PBE and HSE06, fully relativistic norm-conserving PBE pseudopotentials for PBE+SOC preconvergence and HSE06+SOC, and scalar-relativistic RRKJ ultrasoft PBE pseudopotentials for density-functional perturbation-theory phonons. The reference valence configurations, and valence-electron counts are listed in Table S21. All workflows used ecutwfc = 50 Ry and ecutrho = 500 Ry. Self-consistent PBE, HSE06, and HSE06+SOC electronic-structure calculations used a 4 × 4 × 4 Monkhorst–Pack k-point mesh. Band energies were referenced to the Fermi level. Phonons were calculated by density-functional perturbation theory with the Quantum ESPRESSO PHonon package using a 6 × 6 × 6 electronic k-point mesh and a uniform 3 × 3 × 3 q-point mesh. Because this is a reciprocal-space DFPT workflow, a finite-displacement real-space supercell is not applicable. The dynamical matrices were Fourier-interpolated with matdyn.x along L–Γ–X–W. No imaginary modes were observed along this sampled path, but instabilities at unsampled wavevectors elsewhere in the Brillouin zone cannot be excluded; complete Brillouin-zone dynamical

stability is therefore not claimed.
We performed additional collinear spin-polarized PBE calculations using pw.x with nspin = 2. For each compound, initial configurations corresponding to low-spin-like (LS-like), intermediate-spin (IS), and high-spin (HS) states were adopted for the Rh or Co sites. Starting_magnetization values of approximately 0.0111, 0.2222, and 0.4444 were used for the nine-valence-electron Rh and Co norm-conserving pseudopotentials; all other atomic species were initialized with zero magnetization. No total-magnetization constraint was applied. For all three compounds, the nominal IS and HS initializations converged to magnetically equivalent, spin-reversed or nearly identical weak-moment solutions within numerical resolution. The LS-like solutions were higher by 1.901, 2.245, and 5.718 meV per formula unit for Cs_2NaRhF_6, Rb_2NaRhF_6, and Rb_2CoAgF_6, respectively. The revised SI reports the signed total cell moments and total energies in Table S22. We now describe LS, IS, and HS as initialization labels rather than retained final spin states.
Because the primitive cell contains one Rh or Co site, these calculations compare on-site collinear spin initializations and their spin-reversed counterparts; they do not constitute an exhaustive search of long-range antiferromagnetic order, which would require enlarged cells. This scope is now stated explicitly. Since this article does not focus on the study of structural magnetic moments, we have not explored in-depth theoretical calculations.

Data and Code Availability

Data Availability Statement: The processed datasets and code supporting the findings of this study are openly available at https://github.com/qilinwei0/Machine-Learning-of-Band-Gaps-and-Thermodynamic-Stability-in-A2BB-X6-X-F-Cl-Br-I-Double-Perovskites.(Accessed on 22 September 2026) The archive contains the task-specific modeling tables, feature manifest, canonical-formula split assignments, frozen validation and test predictions, candidate-screening results, supplementary sensitivity and distribution-shift outputs, executable scripts, and software requirements. The underlying Materials Project records can be retrieved using the Materials Project identifiers included in the archived tables.

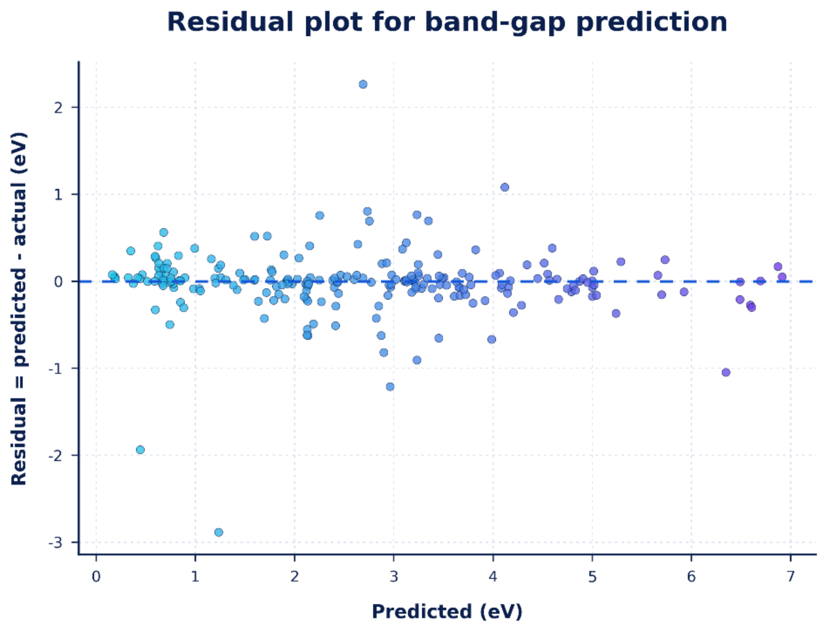

Figure S1. Residuals for held-out band-gap prediction. Residuals are broadly centered on zero, but a small number of high-error samples create a heavy tail.

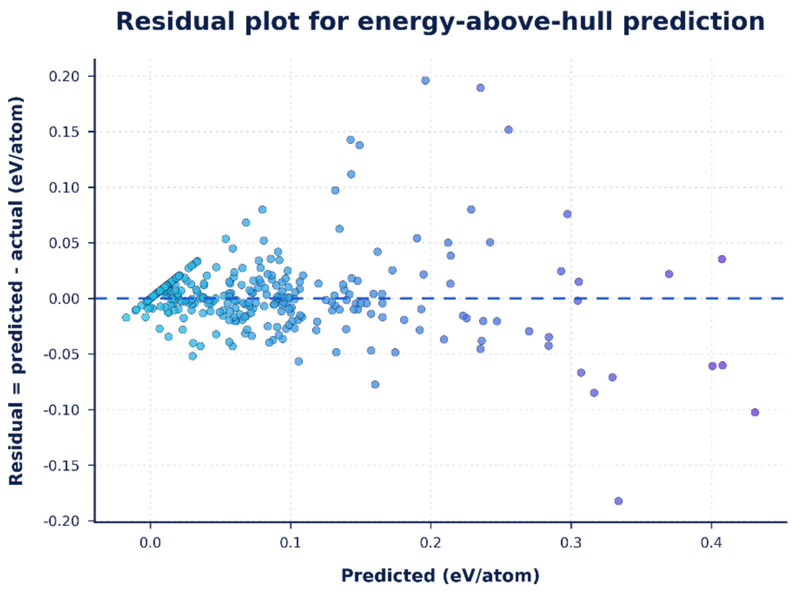

Figure S2. Residuals for held-out energy-above-hull prediction. Dispersion increases with predicted energy, indicating moderate heteroscedasticity outside the near-hull region.

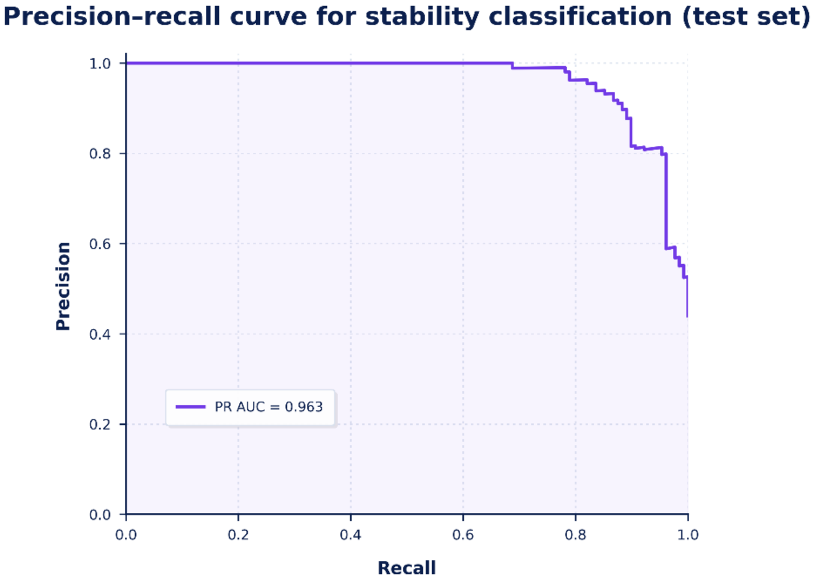

Figure S3. Precision-recall curve for held-out stability classification. The test PR-AUC is 0.9632, substantially exceeding the positive-class prevalence.

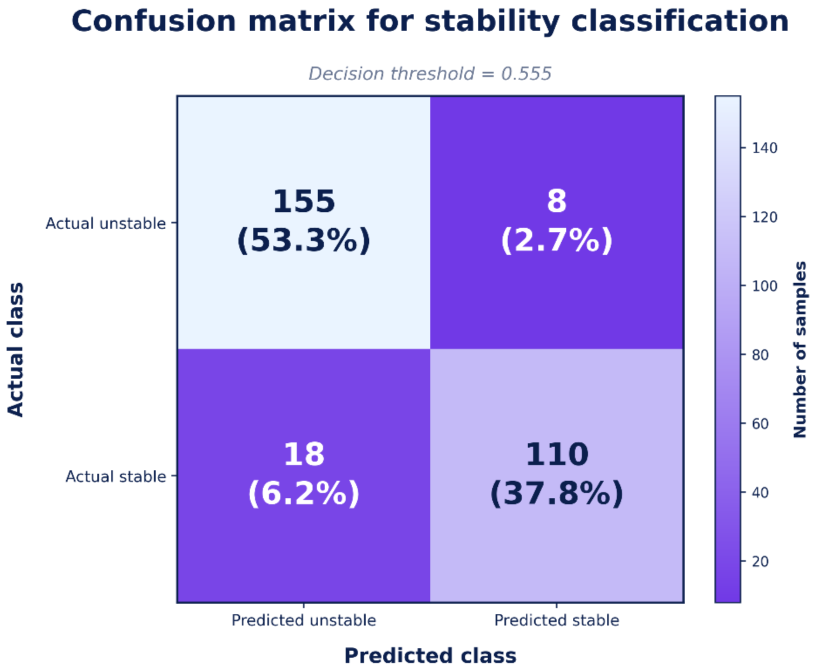

Figure S4. Confusion matrix for stability classification at the validation-selected threshold of 0.555. The matrix contains 155 true negatives, 110 true positives, 8 false positives, and 18 false negatives.

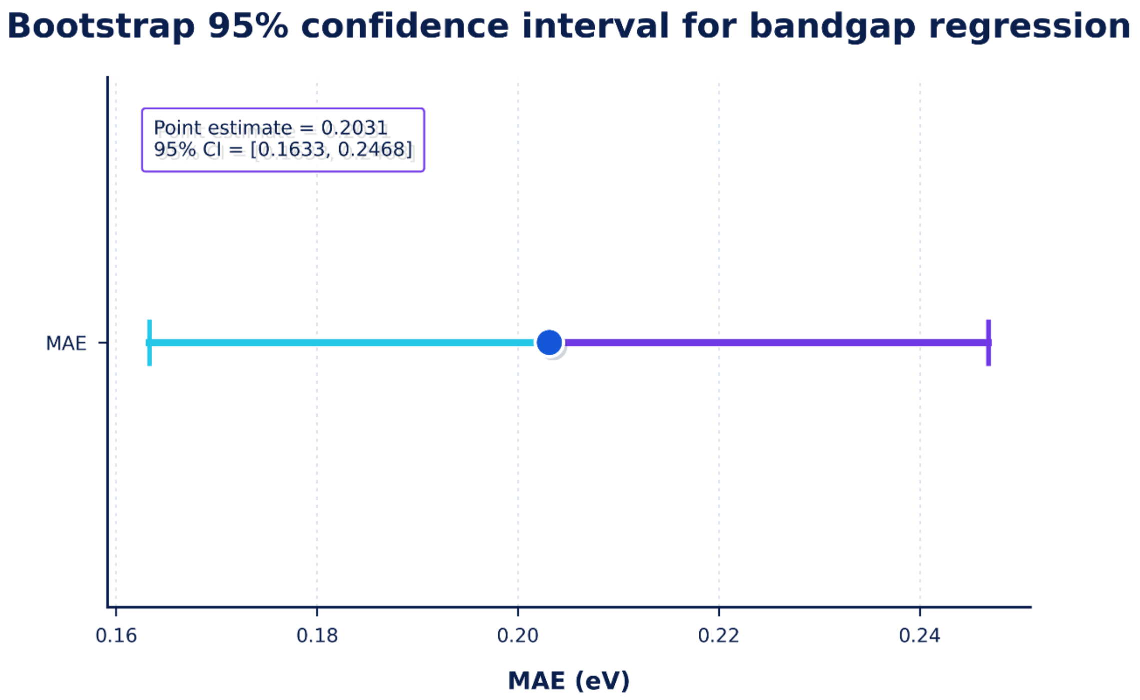

Figure S5. Bootstrap 95% confidence interval for held-out band-gap MAE. The point estimate is 0.2031 eV and the percentile interval is 0.1633-0.2468 eV.

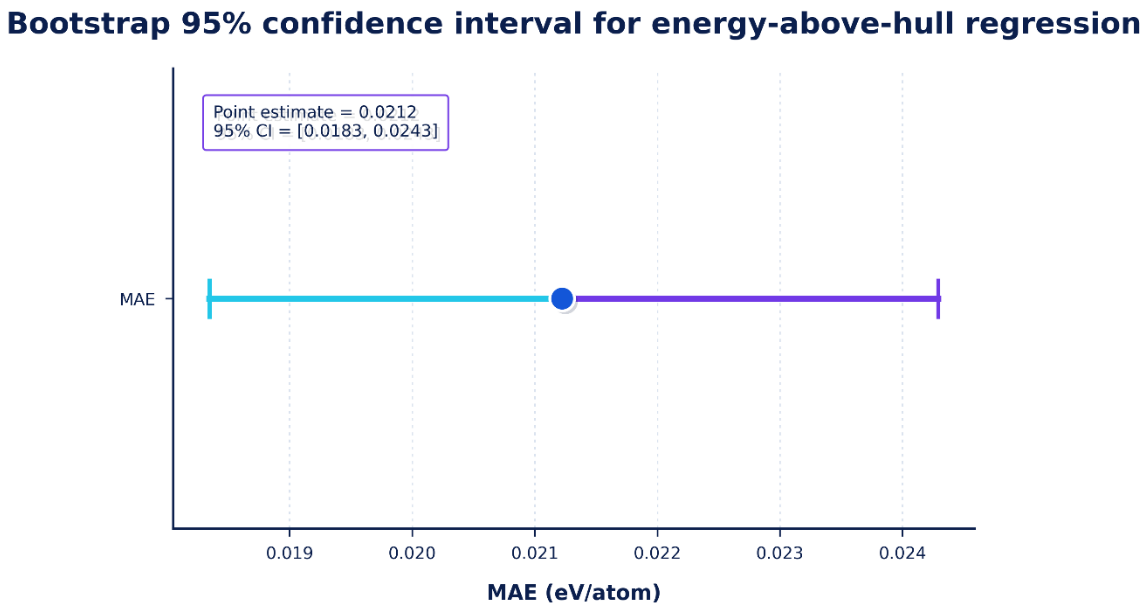

Figure S6. Bootstrap 95% confidence interval for held-out energy-above-hull MAE. The point estimate is 0.02122 eV atom^{-1} and the percentile interval is 0.01835–0.02429 eV atom^{-1}.

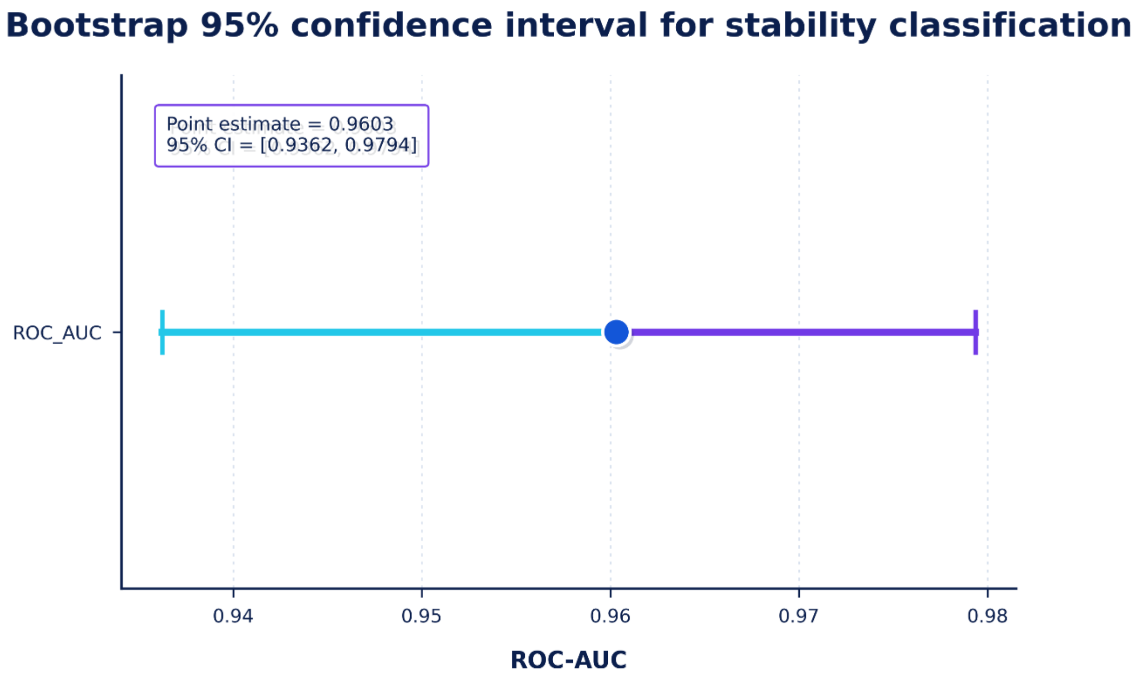

Figure S7. Bootstrap 95% confidence interval for held-out stability ROC-AUC. The point estimate is 0.9603 and the percentile interval is 0.9362-0.9794.

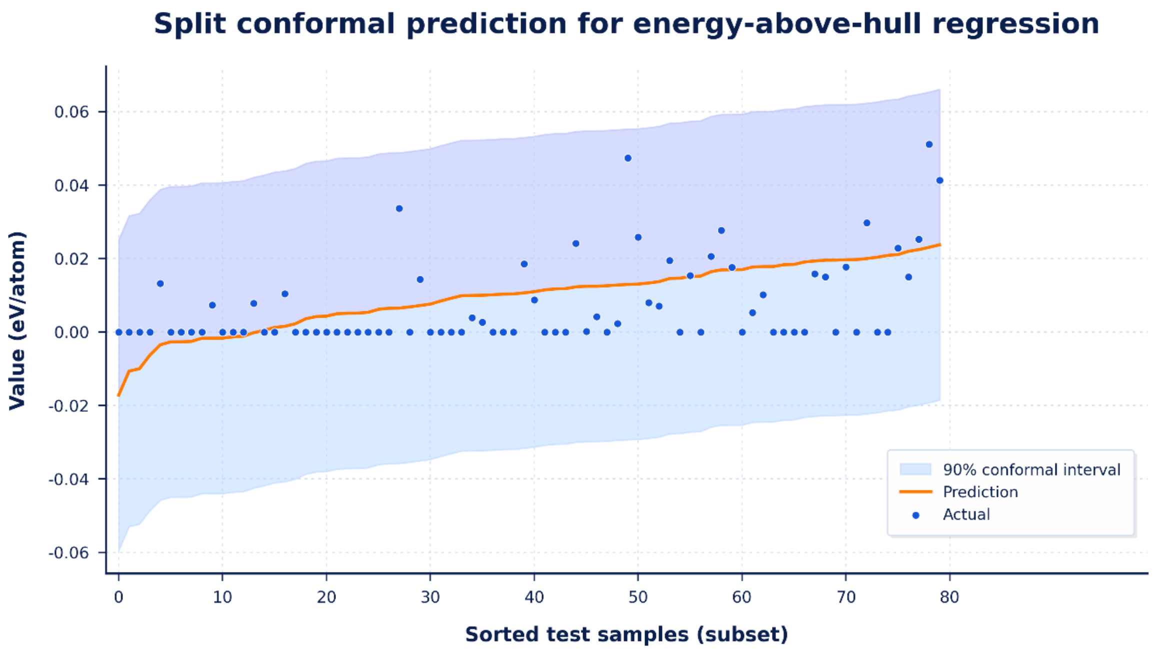

Figure S8. Split-conformal 90% prediction intervals for held-out energy-above-hull regression. The empirical coverage is 87.97% and the average interval width is 0.08456 eV atom^{-1}.

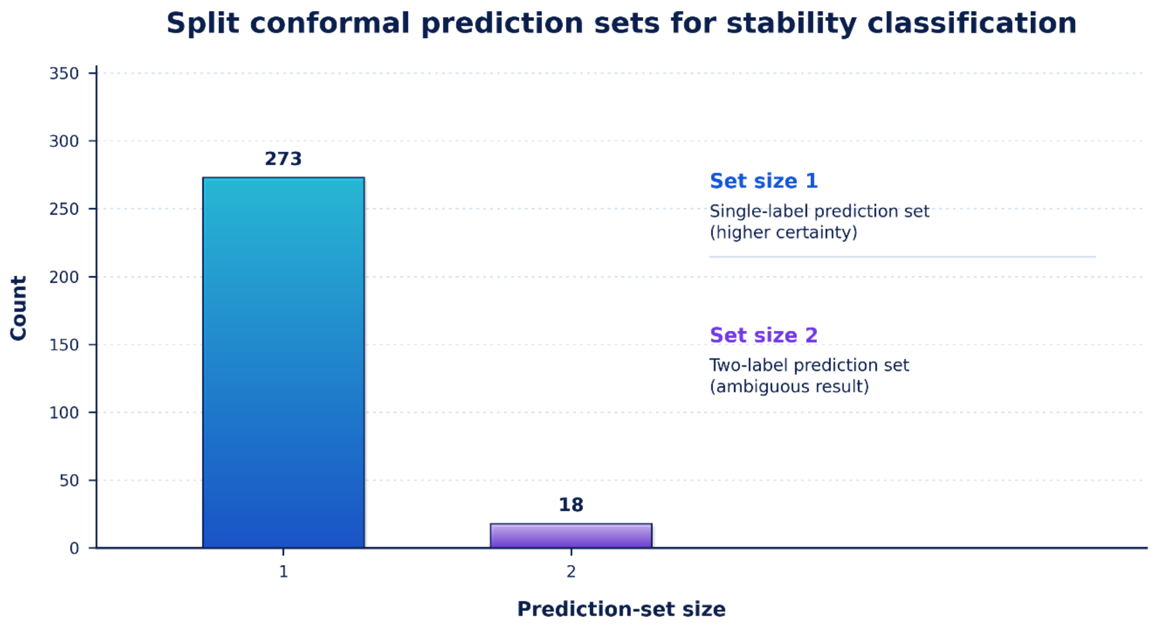

Figure S9. Distribution of split-conformal prediction-set sizes for stability classification. A total of 273 samples receive singleton sets and 18 receive two-label sets.

Table S1. Task-specific datasets, fixed canonical-formula splits, and data-quality statistics.

Task	Samples	Features	Train	Validation	Test	Features with missing values	Max missing rate	Constant features in train
**Bandgap regression**	1418	398	974	221	223	8	44.99 %	20
**Energy-above-hull regression**	1961	398	1373	297	291	8	34.37 %	6
**Stability classification**	1961	398	1373	297	291	8	34.37 %	6

Table S2. Full validation and held-out test comparison of all benchmark models.

Task	Model	Split	MAE	RMSE	R^2	ROC-AUC	PR-AUC	F1	Balanced accuracy	MCC	Threshold
**Bandgap regression**	Mean baseline	validation	1.3842	1.7191	-0.0100	—	—	—	—	—	—
**Bandgap regression**	Mean baseline	test	1.3438	1.6507	-0.0002	—	—	—	—	—	—
**Bandgap regression**	Ridge	validation	0.4883	0.7709	0.7969	—	—	—	—	—	—
**Bandgap regression**	Ridge	test	0.4227	0.5767	0.8779	—	—	—	—	—	—
**Bandgap regression**	Extra Trees	validation	0.1880	0.3855	0.9492	—	—	—	—	—	—
**Bandgap regression**	Extra Trees	test	0.2031	0.3915	0.9437	—	—	—	—	—	—
**Bandgap regression**	HistGB	validation	0.2809	0.4576	0.9284	—	—	—	—	—	—
**Bandgap regression**	HistGB	test	0.2492	0.3772	0.9478	—	—	—	—	—	—
**Energy-above-hull regression**	Mean baseline	validation	0.0625	0.0823	-0.0016	—	—	—	—	—	—
**Energy-above-hull regression**	Mean baseline	test	0.0679	0.0949	-0.0000	—	—	—	—	—	—
**Energy-above-hull regression**	Ridge	validation	0.0267	0.0441	0.7129	—	—	—	—	—	—
**Energy-above-hull regression**	Ridge	test	0.0306	0.0512	0.7088	—	—	—	—	—	—
**Energy-above-hull regression**	Extra Trees	validation	0.0218	0.0380	0.7871	—	—	—	—	—	—
**Energy-above-hull regression**	Extra Trees	test	0.0227	0.0426	0.7983	—	—	—	—	—	—
**Energy-above-hull**	HistGB	validation	0.0184	0.0277	0.8864	—	—	—	—	—	—

**regression**											
**Energy-above-hull regression**	HistGB	test	0.0212	0.0355	0.8601	—	—	—	—	—	—
**Stability classification**	Majority baseline	validation	—	—	—	0.5000	0.4242	0.5957	0.5000	0.0000	0.050
**Stability classification**	Majority baseline	test	—	—	—	0.5000	0.4399	0.6110	0.5000	0.0000	0.050
**Stability classification**	Logistic regression	validation	—	—	—	0.9163	0.8562	0.8487	0.8686	0.7290	0.460
**Stability classification**	Logistic regression	test	—	—	—	0.9219	0.9316	0.8828	0.8954	0.7908	0.460
**Stability classification**	Extra Trees	validation	—	—	—	0.9399	0.9169	0.8571	0.8764	0.7452	0.430
**Stability classification**	Extra Trees	test	—	—	—	0.9551	0.9534	0.8669	0.8809	0.7582	0.430
**Stability classification**	HistGB	validation	—	—	—	0.9472	0.9086	0.8745	0.8906	0.7857	0.555
**Stability classification**	HistGB	test	—	—	—	0.9603	0.9632	0.8943	0.9051	0.8192	0.555

Table S3. Feature-ablation results for all, non-structural, and structural-only descriptor sets.

Task	Feature set	Features	Validation metric	Test metric	Secondary test metric
**Bandgap regression**	All features	398	MAE 0.18802; R^2 0.9492	MAE 0.20310	R^2 0.9437
**Bandgap regression**	Non-structural features	359	MAE 0.18786; R^2 0.9478	MAE 0.20435	R^2 0.9422
**Bandgap regression**	Structural features only	39	MAE 1.01834; R^2 0.3469	MAE 0.94736	R^2 0.3949
**Energy-above-hull regression**	All features	398	MAE 0.01844; R^2 0.8864	MAE 0.02122	R^2 0.8601
**Energy-above-hull regression**	Non-structural features	359	MAE 0.01958; R^2 0.8748	MAE 0.02388	R^2 0.8156

**Energy-above-hull regression**	Structural features only	39	MAE 0.03897; R^2 0.5760	MAE 0.04386	R^2 0.5263
**Stability classification**	All features	398	ROC-AUC 0.9472; MCC 0.7857	ROC-AUC 0.9603	MCC 0.8192
**Stability classification**	Non-structural features	359	ROC-AUC 0.9390; MCC 0.7761	ROC-AUC 0.9522	MCC 0.7786
**Stability classification**	Structural features only	39	ROC-AUC 0.8414; MCC 0.5323	ROC-AUC 0.8326	MCC 0.4948

Table S4. Ten highest validation-set permutation importances for each selected model.

Task	Rank	Feature	Permutation importance
**Band-gap regression**	1	phys·delta·chi·BX·mean	0.134154
**Band-gap regression**	2	B·max·d·valence	0.126662
**Band-gap regression**	3	phys·B·d·valence·mean	0.070884
**Band-gap regression**	4	B·mean·d·valence	0.063281
**Band-gap regression**	5	orig·avg·d·valence·electrons	0.062588
**Band-gap regression**	6	B·mean·pauling·en	0.057201
**Band-gap regression**	7	B·mean·outer·electrons	0.055877
**Band-gap regression**	8	B·max·mendeleev·number	0.045516
**Band-gap regression**	9	B·absdiff·group	0.041480
**Band-gap regression**	10	comp·Ce·fraction	0.038621
**Energy-above-hull regression**	1	struct·AX·length·over·covalent·rsum	0.018443
**Energy-above-hull regression**	2	phys·mass·ratio·A·to·Bmean	0.007181
**Energy-above-hull regression**	3	B·mean·first·ip·eV	0.004955
**Energy-above-hull regression**	4	A·atomic·mass	0.004306
**Energy-above-hull regression**	5	phys·B·oxidation·mean	0.002815
**Energy-above-hull regression**	6	phys·BX·radius·ratio	0.002803
**Energy-above-hull regression**	7	B·max·metallic·radius·A	0.002190
**Energy-above-hull regression**	8	struct·lattice·a·A	0.001451
**Energy-above-hull regression**	9	B·min·polarizability·1e24cm3	0.001345
**Energy-above-hull regression**	10	B·max·pauling·en	0.001313
**Stability classification**	1	struct·AX·length·over·covalent·rsum	0.053792
**Stability classification**	2	phys·mass·ratio·A·to·Bmean	0.031792
**Stability classification**	3	B·min·covalent·radius·A	0.014017
**Stability classification**	4	struct·mean·shell·gap·ratio	0.005848
**Stability classification**	5	phys·BX·radius·ratio	0.005662
**Stability classification**	6	B·min·atomic·radius·A	0.004270
**Stability classification**	7	B·max·pearson·hardness·eV	0.004084
**Stability classification**	8	struct·BX·length·over·covalent·rsum	0.004038

**Stability classification**	9	B·absdiff·pauling·en	0.003527
**Stability classification**	10	B·mean·first·ip·eV	0.003527

Table S5. Repeated formula-grouped cross-validation summary.

Task	Metric	Mean	SD	2.5th percentile	97.5th percentile	Folds
**Bandgap regression**	MAE	0.21130	0.01788	0.19070	0.24805	12
**Bandgap regression**	RMSE	0.43037	0.05224	0.36766	0.52963	12
**Bandgap regression**	R2	0.93203	0.01918	0.89590	0.95551	12
**Energy-above-hull regression**	MAE	0.02172	0.00231	0.01812	0.02534	12
**Energy-above-hull regression**	RMSE	0.03905	0.00783	0.02882	0.05234	12
**Energy-above-hull regression**	R2	0.81405	0.06059	0.70604	0.88036	12
**Stability classification**	ROC_AUC	0.95755	0.00883	0.94671	0.97060	12
**Stability classification**	PR_AUC	0.94754	0.01475	0.92427	0.96801	12
**Stability classification**	F1	0.87599	0.01477	0.85484	0.89770	12
**Stability classification**	Balanced_Accuracy	0.88847	0.01098	0.87497	0.90525	12
**Stability classification**	MCC	0.77448	0.02310	0.74550	0.80829	12

Table S6. Bootstrap percentile confidence intervals for held-out test metrics.

Task	Metric	Point estimate	CI lower	CI upper	Bootstrap resamples
**Bandgap regression**	MAE	0.203103	0.163286	0.246805	500
**Bandgap regression**	RMSE	0.391488	0.274185	0.506692	500
**Bandgap regression**	R2	0.943742	0.899738	0.971721	500
**Energy-above-hull regression**	MAE	0.021223	0.018347	0.024290	500
**Energy-above-hull regression**	RMSE	0.035500	0.028652	0.042308	500
**Energy-above-hull regression**	R2	0.860105	0.788535	0.905867	500
**Stability classification**	ROC_AUC	0.960314	0.936228	0.979352	500
**Stability classification**	PR_AUC	0.963169	0.939703	0.980635	500
**Stability classification**	F1	0.894309	0.855892	0.934644	500
**Stability classification**	Balanced_Accuracy	0.905148	0.869197	0.937873	500
**Stability classification**	MCC	0.819178	0.749460	0.878349	500

Table S7. Split-conformal calibration and held-out coverage summary.

Task	Method	Nominal coverage	Calibration quantile	Empirical coverage	Average width / set size	Samples
**Bandgap regression**	split conformal interval	0.90	0.434395	0.8834	0.868789	223
**Energy-above-hull regression**	split conformal interval	0.90	0.042278	0.8797	0.084556	291
**Stability classification**	split conformal set	0.90	0.663977	0.9313	1.061856	291

Table S8. Held-out samples satisfying the point criteria, reported without a weighted composite score. Reference labels are shown only for retrospective evaluation.

Formula	MP-ID	Pred. gap	Pred. E_{hull}	P(stable)	MP-Ref. gap	MP-Ref. E_{hull}	MP-Ref. stable	Model–MP hit
**$Rb_2InAgCl_6$**	mp-1111634	1.308	0.0004	0.991	1.293	0.0000	Yes	Yes
**$Cs_2NaCrCl_6$**	mp-1205696	1.447	0.0133	0.996	1.353	0.0080	Yes	Yes
**Cs_2KCeBr_6**	mp-1112787	1.724	0.0036	0.999	1.206	0.0000	Yes	Yes
**$Cs_2LiInBr_6$**	mp-989405	1.600	0.0203	0.996	1.579	0.0000	Yes	Yes
**Rb_2NaRhF_6**	mp-14038	1.928	-0.0107	0.998	1.957	0.0000	Yes	Yes
**Cs_2AgBiI_6**	mp-1113264	1.382	0.0200	0.976	1.428	0.0297	Yes	Yes
**Cs_2KMoCl_6**	mp-1111876	1.773	0.0110	0.991	1.666	0.0087	Yes	Yes
**$Cs_2NaTlCl_6$**	mp-1112527	1.874	0.0062	0.996	1.918	0.0000	Yes	Yes
**$Cs_2TlSbBr_6$**	mp-1112108	1.205	0.0195	0.998	1.225	0.0150	Yes	Yes
**$Cs_2RbCeCl_6$**	mp-1112520	1.235	0.0237	0.994	4.120	0.0413	Yes	No
**Cs_2NaRhF_6**	mp-1111979	1.933	0.0196	0.998	1.908	0.0000	Yes	Yes
**$Rb_2TlAsCl_6$**	mp-1114009	1.943	0.0178	0.909	1.954	0.0101	Yes	Yes
**$K_2NaTlCl_6$**	mp-1111616	1.841	0.0378	0.965	1.989	0.0368	Yes	Yes
**$Rb_2NaInBr_6$**	mp-1114017	1.975	0.0247	0.913	2.005	0.0270	Yes	No
**Rb_2CoAgF_6**	mp-1110611	1.790	0.0470	0.778	2.009	0.0189	Yes	No
**Cs_2LiSbI_6**	mp-1112673	1.931	0.0354	0.743	1.925	0.0355	Yes	Yes
**$K_2AgSbBr_6$**	mp-1110912	1.040	0.0403	0.703	1.125	0.0197	Yes	Yes

Table S9. Descriptors containing missing values in each task-specific dataset.

Task	Feature	Missing count	Missing rate

**Bandgap regression**	phys·B·d0·or·d10·count	638	44.993%
**Bandgap regression**	phys·B·d·after·ox·mean	638	44.993%
**Bandgap regression**	phys·B·oxidation·absdiff	14	0.987%
**Bandgap regression**	phys·B·oxidation·max	14	0.987%
**Bandgap regression**	phys·B·oxidation·mean	14	0.987%
**Bandgap regression**	phys·B·oxidation·min	14	0.987%
**Bandgap regression**	phys·heterovalent·B·pair	14	0.987%
**Bandgap regression**	phys·oxidation·charge·residual	14	0.987%
**Energy-above-hull regression**	phys·B·d0·or·d10·count	674	34.370%
**Energy-above-hull regression**	phys·B·d·after·ox·mean	674	34.370%
**Energy-above-hull regression**	phys·B·oxidation·absdiff	15	0.765%
**Energy-above-hull regression**	phys·B·oxidation·max	15	0.765%
**Energy-above-hull regression**	phys·B·oxidation·mean	15	0.765%
**Energy-above-hull regression**	phys·B·oxidation·min	15	0.765%
**Energy-above-hull regression**	phys·heterovalent·B·pair	15	0.765%
**Energy-above-hull regression**	phys·oxidation·charge·residual	15	0.765%
**Stability classification**	phys·B·d0·or·d10·count	674	34.370%
**Stability classification**	phys·B·d·after·ox·mean	674	34.370%
**Stability classification**	phys·B·oxidation·absdiff	15	0.765%
**Stability classification**	phys·B·oxidation·max	15	0.765%
**Stability classification**	phys·B·oxidation·mean	15	0.765%
**Stability classification**	phys·B·oxidation·min	15	0.765%
**Stability classification**	phys·heterovalent·B·pair	15	0.765%
**Stability classification**	phys·oxidation·charge·residual	15	0.765%

Table S10. Independent-refit sensitivity to sparse-descriptor removal on the fixed test set. Values are compared with the matching all-feature baseline from the same refit; the frozen prediction files remain authoritative for the principal fixed-test metrics.

Task	Variant	Features	Dropped	Performance
bandgap	independent-refit all-feature baseline: all 398 with train median and indicators	398	0	MAE 0.20127; R^2 0.9432

bandgap	drop features over 20pct missing	396	2	MAE 0.20304; R^2 0.9416
bandgap	drop every feature with any training missingness	390	8	MAE 0.20219; R^2 0.9428
hull	independent-refit all-feature baseline: all 398 with train median and indicators	398	0	MAE 0.02101; R^2 0.8544
hull	drop features over 20pct missing	396	2	MAE 0.02123; R^2 0.8484
hull	drop every feature with any training missingness	390	8	MAE 0.02094; R^2 0.8489
stability	independent-refit all-feature baseline: all 398 with train median and indicators	398	0	ROC-AUC 0.9607; MCC 0.8192
stability	drop features over 20pct missing	396	2	ROC-AUC 0.9604; MCC 0.8265
stability	drop every feature with any training missingness	390	8	ROC-AUC 0.9572; MCC 0.8119

Table S11. Expanded algorithm benchmark on the unchanged validation split.

Task	Model	Validation performance
bandgap	mean baseline	MAE 1.38416; R^2 -0.0100
bandgap	ridge	MAE 0.52014; R^2 0.7508
bandgap	svr rbf	MAE 0.20862; R^2 0.8938
bandgap	random forest	MAE 0.25263; R^2 0.9358
bandgap	extra trees	MAE 0.18671; R^2 0.9508
bandgap	hist gradient boosting	MAE 0.27468; R^2 0.9301
hull	mean baseline	MAE 0.06251; R^2 -0.0016
hull	ridge	MAE 0.02678; R^2 0.6413
hull	svr rbf	MAE 0.03104; R^2 0.7331
hull	random forest	MAE 0.02012; R^2 0.8641
hull	extra trees	MAE 0.02161; R^2 0.7980
hull	hist gradient boosting	MAE 0.01799; R^2 0.8875
stability	majority baseline	ROC-AUC 0.5000; MCC 0.0000
stability	logistic regression	ROC-AUC 0.9171; MCC 0.7152
stability	svc rbf	ROC-AUC 0.9483; MCC 0.7395

stability	random forest	ROC-AUC 0.9408; MCC 0.7582
stability	extra trees	ROC-AUC 0.9393; MCC 0.7185
stability	hist gradient boosting	ROC-AUC 0.9492; MCC 0.7718

Table S12. Bounded post-hoc hyperparameter robustness on the validation split.

Task	Setting	Performance
bandgap	trees=200, max_features=0.5	MAE 0.18380
bandgap	trees=200, max_features=0.7	MAE 0.18841
bandgap	trees=200, max_features=1.0	MAE 0.19030
bandgap	trees=400, max_features=0.5	MAE 0.18182
bandgap	trees=400, max_features=0.7	MAE 0.18671
bandgap	trees=400, max_features=1.0	MAE 0.19032
bandgap	trees=800, max_features=0.5	MAE 0.18061
bandgap	trees=800, max_features=0.7	MAE 0.18523
bandgap	trees=800, max_features=1.0	MAE 0.18995
hull	lr=0.03, leaves=15	MAE 0.01829
hull	lr=0.03, leaves=31	MAE 0.01793
hull	lr=0.05, leaves=15	MAE 0.01799
hull	lr=0.05, leaves=31	MAE 0.01782
hull	lr=0.10, leaves=15	MAE 0.01747
hull	lr=0.10, leaves=31	MAE 0.01787
stability	lr=0.03, leaves=15	ROC-AUC 0.9461; MCC 0.7648
stability	lr=0.03, leaves=31	ROC-AUC 0.9452; MCC 0.7579
stability	lr=0.05, leaves=15	ROC-AUC 0.9492; MCC 0.7718
stability	lr=0.05, leaves=31	ROC-AUC 0.9463; MCC 0.7376
stability	lr=0.10, leaves=15	ROC-AUC 0.9467; MCC 0.7720
stability	lr=0.10, leaves=31	ROC-AUC 0.9433; MCC 0.7376

Table S13. Fixed-test performance by halide family.

Task	Halide	n	Performance
bandgap	Br	38	MAE 0.10906; R^2 0.9779
bandgap	Cl	67	MAE 0.15591; R^2 0.9140
bandgap	F	88	MAE 0.26507; R^2 0.9624
bandgap	I	30	MAE 0.24586; R^2 0.7262
hull	Br	49	MAE 0.01284; R^2 0.9203
hull	Cl	83	MAE 0.00967; R^2 0.9375
hull	F	126	MAE 0.03437; R^2 0.8337
hull	I	33	MAE 0.01253; R^2 0.8268
stability	Br	49	ROC-AUC 0.9552; MCC 0.6880
stability	Cl	83	ROC-AUC 0.9959; MCC 0.9518

stability	F	126	ROC-AUC 0.9383; MCC 0.7689
stability	I	33	ROC-AUC 0.9596; MCC 0.8199

Table S14. Stronger chemical-distribution-shift tests. Clusters were defined without target labels from the 359 non-structural descriptors.

Task	Held-out family/cluster	n	Performance
bandgap	leave out Br	231	MAE 0.27362; R^2 0.9066
bandgap	leave out Cl	458	MAE 0.47130; R^2 0.8068
bandgap	leave out F	537	MAE 1.08550; R^2 0.5433
bandgap	leave out I	192	MAE 0.63277; R^2 0.3195
hull	leave out Br	317	MAE 0.01522; R^2 0.8767
hull	leave out Cl	567	MAE 0.02752; R^2 0.5097
hull	leave out F	829	MAE 0.05897; R^2 0.4328
hull	leave out I	248	MAE 0.02145; R^2 0.6940
stability	leave out Br	317	ROC-AUC 0.9459; MCC 0.6918
stability	leave out Cl	567	ROC-AUC 0.9247; MCC 0.6448
stability	leave out F	829	ROC-AUC 0.8549; MCC 0.5628
stability	leave out I	248	ROC-AUC 0.8879; MCC 0.6036
bandgap	cluster 0	332	MAE 1.29866; R^2 -0.0080
bandgap	cluster 1	337	MAE 1.20077; R^2 0.1733
bandgap	cluster 2	410	MAE 1.03926; R^2 -0.3499
bandgap	cluster 3	325	MAE 0.70147; R^2 0.1381
bandgap	cluster 4	14	MAE 1.17793; R^2 -1.6578
hull	cluster 0	424	MAE 0.04530; R^2 0.0527
hull	cluster 1	32	MAE 0.28502; R^2 -2.2111
hull	cluster 2	415	MAE 0.06090; R^2 0.4669
hull	cluster 3	410	MAE 0.06095; R^2 0.3623
hull	cluster 4	680	MAE 0.02797; R^2 0.5052
stability	cluster 0	424	ROC-AUC 0.7872; MCC 0.3943
stability	cluster 1	32	ROC-AUC 0.7344; MCC 0.0000
stability	cluster 2	415	ROC-AUC 0.8525; MCC 0.5389
stability	cluster 3	410	ROC-AUC 0.9353; MCC 0.7047
stability	cluster 4	680	ROC-AUC 0.8722; MCC 0.5924

Table S15. Fixed-test performance by training frequency of the B/B′–X combination.

Task	Training-frequency bin	n	Performance
bandgap	0-2 (rare)	196	MAE 0.19818; R^2 0.9418
bandgap	3-5	27	MAE 0.23884; R^2 0.9551

hull	0-2 (rare)	253	MAE 0.02115; R^2 0.8543
hull	3-5	37	MAE 0.02116; R^2 0.8779
hull	6-10	1	MAE 0.04249; R^2 —
stability	0-2 (rare)	253	ROC-AUC 0.9543; MCC 0.8076
stability	3-5	37	ROC-AUC 1.0000; MCC 0.8956
stability	6-10	1	ROC-AUC —; MCC 0.0000

Table S16. E_{hull} target-skew sensitivity on the fixed test set (matched post-hoc rerun).

Variant	Subset	n	MAE	R^2
untransformed selected model	all	291	0.02081	0.8547
untransformed selected model	near hull le 0.10	203	0.01695	0.0372
untransformed selected model	tail gt 0.10	88	0.02972	0.8143
log1p target scale 0.01	all	291	0.01973	0.8871
log1p target scale 0.01	near hull le 0.10	203	0.01425	0.5068
log1p target scale 0.01	tail gt 0.10	88	0.03238	0.7887
yeo johnson target	all	291	0.02102	0.8554
yeo johnson target	near hull le 0.10	203	0.01474	0.4087
yeo johnson target	tail gt 0.10	88	0.03553	0.7186
tail weight 3x above 0.10	all	291	0.02245	0.8413
tail weight 3x above 0.10	near hull le 0.10	203	0.01883	-0.1059
tail weight 3x above 0.10	tail gt 0.10	88	0.03080	0.8116

Table S17. Joint descriptor-family permutation importance on the validation split (10 repeats). Positive values indicate MAE increase or ROC-AUC decrease.

Task	Descriptor family	Features	Metric	Mean importance	SD
bandgap	structural descriptors	39	MAE	0.07842	0.00768
bandgap	composition and site indicators	79	MAE	0.12699	0.00988
bandgap	crystallographic indicators	23	MAE	-0.00006	0.00015
bandgap	oxidation and	36	MAE	0.37234	0.01579

	physics interactions				
bandgap	elemental and exchange-invariant properties	221	MAE	1.26754	0.04752
hull	structural descriptors	39	MAE	0.02434	0.00108
hull	composition and site indicators	79	MAE	0.00027	0.00009
hull	crystallographic indicators	23	MAE	0.00000	0.00000
hull	oxidation and physics interactions	36	MAE	0.01780	0.00183
hull	elemental and exchange-invariant properties	221	MAE	0.03373	0.00191
stability	structural descriptors	39	ROC-AUC	0.11837	0.01735
stability	composition and site indicators	79	ROC-AUC	-0.00016	0.00058
stability	crystallographic indicators	23	ROC-AUC	0.00000	0.00000
stability	oxidation and physics interactions	36	ROC-AUC	0.05418	0.00835
stability	elemental and exchange-invariant properties	221	ROC-AUC	0.17371	0.00832

Table S18. Exact inference for empirical coverage relative to the nominal 90% level.

Task	Covered/n	Coverage	Exact 95% CI	Two-sided p
bandgap	197/223	88.34%	83.38–92.24%	0.433
hull	256/291	87.97%	83.67–91.48%	0.241
stability	271/291	93.13%	89.58–95.75%	0.078

Table S19. Uncertainty-tiered retrospective prioritization of the 17 held-out samples passing

point criteria. The reference-hit column is used only for post-hoc evaluation.

Formula	MP-ID	Pred. gap	Pred. Ehull	P(stable)	90% Ehull interval	Set	Tier	Ref. hit
Rb_2NaRhF_6	mp-14038	1.928	-0.0107	0.998	[-0.0529, 0.0316]	{stable}	Tier 1	Yes
$Rb_2InAgCl_6$	mp-1111634	1.308	0.0004	0.991	[-0.0419, 0.0427]	{stable}	Tier 1	Yes
Cs_2KCeBr_6	mp-1112787	1.724	0.0036	0.999	[-0.0387, 0.0459]	{stable}	Tier 1	Yes
$Cs_2NaTlCl_6$	mp-1112527	1.874	0.0062	0.996	[-0.0361, 0.0484]	{stable}	Tier 1	Yes
Cs_2KMoCl_6	mp-1111876	1.773	0.0110	0.991	[-0.0313, 0.0532]	{stable}	Tier 2	Yes
$Cs_2NaCrCl_6$	mp-1205696	1.447	0.0133	0.996	[-0.0289, 0.0556]	{stable}	Tier 2	Yes
$Rb_2TlAsCl_6$	mp-1114009	1.943	0.0178	0.909	[-0.0245, 0.0601]	{stable}	Tier 2	Yes
$Cs_2TlSbBr_6$	mp-1112108	1.205	0.0195	0.998	[-0.0227, 0.0618]	{stable}	Tier 2	Yes
Cs_2NaRhF_6	mp-1111979	1.933	0.0196	0.998	[-0.0227, 0.0618]	{stable}	Tier 2	Yes
Cs_2AgBiI_6	mp-1113264	1.382	0.0200	0.976	[-0.0223, 0.0623]	{stable}	Tier 2	Yes
$Cs_2LiInBr_6$	mp-989405	1.600	0.0203	0.996	[-0.0219, 0.0626]	{stable}	Tier 2	Yes
$Cs_2RbCeCl_6$	mp-1112520	1.235	0.0237	0.994	[-0.0185, 0.0660]	{stable}	Tier 2	No
$Rb_2NaInBr_6$	mp-1114017	1.975	0.0247	0.913	[-0.0176,	{stable}	Tier 2	No

					0.0670]			
Cs_2LiSbI_6	mp-1112673	1.931	0.0354	0.743	[-0.0069, 0.0776]	{stable}	Tier 2	Yes
$K_2NaTlCl_6$	mp-1111616	1.841	0.0378	0.965	[-0.0045, 0.0800]	{stable}	Tier 2	Yes
$K_2AgSbBr_6$	mp-1110912	1.040	0.0403	0.703	[-0.0020, 0.0825]	{stable}	Tier 2	Yes
Rb_2CoAgF_6	mp-1110611	1.790	0.0470	0.778	[0.0047, 0.0892]	{stable}	Tier 2	No

Table S20. Independent first-principles electronic validation and path-restricted phonon results for one Tier-1 candidate and two representative Tier-2 shortlisted fluorides.

Compound	Tier	MP gap (eV)	PBE (eV)	HSE06 (eV)	HSE06+SOC (eV)	Phonons
Cs_2NaRhF_6	Tier 2	1.908	1.71	2.85	2.849	No imaginary mode along sampled L–Γ–X–W path
Rb_2CoAgF_6	Tier 2	2.009	1.18	2.15	2.148	No imaginary mode along sampled L–Γ–X–W path
Rb_2NaRhF_6	Tier 1	1.957	1.75	2.92	2.919	No imaginary mode along sampled L–Γ–X–W path

Table S21. Pseudopotentials used in the electronic-structure and DFPT phonon calculations.

Workflow	Element	Exact filename	Type	Relativity	Reference valence configuration	Zval
PBE and HSE06	Cs	Cs.pbe-nc.UPF	NC	Scalar	$6s^1$	1
PBE and HSE06	Rb	Rb.pbe-nc.UPF	NC	Scalar	$5s^1$	1
PBE and HSE06	Na	Na.pbe-nc.UPF	NC	Scalar	$3s^1$	1
PBE and HSE06	Rh	Rh.pbe-nc.UPF	NC	Scalar	$4d^8 5s^1$	9
PBE and HSE06	Co	Co.pbe-nc.UPF	NC	Scalar	$3d^7 4s^2$	9
PBE and HSE06	Ag	Ag.pbe-nc.UPF	NC	Scalar	$4d^{10} 5s^1$	11

Workflow	Element	Exact filename	Type	Relativity	Reference valence configuration	Zval
PBE and HSE06	F	F.pbe-nc.UPF	NC	Scalar	$2s^2\ 2p^5$	7
PBE+SOC and HSE06+SOC	Cs	Cs.rel-pbe-nc.UPF	NC	Fully relativistic	$6s^1$	1
PBE+SOC and HSE06+SOC	Rb	Rb.rel-pbe-nc.UPF	NC	Fully relativistic	$5s^1$	1
PBE+SOC and HSE06+SOC	Na	Na.rel-pbe-nc.UPF	NC	Fully relativistic	$3s^1$	1
PBE+SOC and HSE06+SOC	Rh	Rh.rel-pbe-nc.UPF	NC	Fully relativistic	$4d^8\ 5s^1$	9
PBE+SOC and HSE06+SOC	Co	Co.rel-pbe-nc.UPF	NC	Fully relativistic	$3d^7\ 4s^2$	9
PBE+SOC and HSE06+SOC	Ag	Ag.rel-pbe-nc.UPF	NC	Fully relativistic	$4d^{10}\ 5s^1$	11
PBE+SOC and HSE06+SOC	F	F.rel-pbe-nc.UPF	NC	Fully relativistic	$2s^2\ 2p^5$	7
DFPT phonons	Cs	Cs.pbe-spn-rrkjus.UPF	RRKJ USPP	Scalar	$5s^2\ 5p^6\ 6s^1$	9
DFPT phonons	Rb	Rb.pbe-spn-rrkjus.UPF	RRKJ USPP	Scalar	$4s^2\ 4p^6\ 5s^1$	9
DFPT phonons	Na	Na.pbe-spn-rrkjus.UPF	RRKJ USPP	Scalar	$2s^2\ 2p^6\ 3s^{0.5}$	9
DFPT phonons	Rh	Rh.pbe-spn-rrkjus.UPF	RRKJ USPP	Scalar	$4s^2\ 4p^6\ 4d^7\ 5s^2$	17
DFPT phonons	Co	Co.pbe-n-rrkjus.UPF	RRKJ USPP	Scalar	$3d^7\ 4s^2$	9
DFPT phonons	Ag	Ag.pbe-rrkjus.UPF	RRKJ USPP	Scalar	$4d^{9.5}\ 5s^{1.5}$	11
DFPT phonons	F	F.pbe-rrkjus.UPF	RRKJ USPP	Scalar	$2s^2\ 2p^5$	7

Table S22. Dependence of collinear spin-polarized PBE solutions on the initial Rh or Co magnetization. nspin = 2 was used without a fixed-total-magnetization constraint.

Compound	Initialization	starting_magnetization	Final cell moment (μB/cell)	Total energy (eV/cell)
Cs_2NaRhF_6	LS-like	0.0111	0.0051	−42.53906551
Cs_2NaRhF_6	IS	0.2222	−0.2115	−42.54096675
Cs_2NaRhF_6	HS	0.4444	0.2119	−42.54096677
Rb_2NaRhF_6	LS-like	0.0111	−0.0239	−43.32489710
Rb_2NaRhF_6	IS	0.2222	−0.2091	−43.32714221
Rb_2NaRhF_6	HS	0.4444	0.2089	−43.32713753
Rb_2CoAgF_6	LS-like	0.0111	0.0020	−41.84428908
Rb_2CoAgF_6	IS	0.2222	0.1637	−41.85000675
Rb_2CoAgF_6	HS	0.4444	−0.1650	−41.85000484

Table S23. Sensitivity of the retained candidate count to the P(stable) threshold while the

predicted band-gap criterion (1.0–2.0 eV) and predicted Ehull criterion (≤ 0.05 eV atom^{-1}) are held fixed.The four Tier-1 candidates remain unchanged across all thresholds because each has P(stable) > 0.99.

P(stable) threshold	Retained candidates
0.50	19
0.60	18
0.65	18
0.70	17
0.75	15
0.80	14
0.90	14

References:

1 . Ward, L.; Dunn, A.; Faghaninia, A.; Zimmermann, N. E. R.; Bajaj, S.; Wang, Q.; Montoya, J.; Chen, J.; Bystrom, K.; Dylla, M.; Chard, K.; Asta, M.; Persson, K. A.; Snyder, G. J.; Foster, I.; Jain, A. Matminer: An open source toolkit for materials data mining. *Computational Materials Science* **2018**, *152*, 60-69.

2 . Jain, A.; Ong, S. P.; Hautier, G.; Chen, W.; Richards, W. D.; Dacek, S.; Cholia, S.; Gunter, D.; Skinner, D.; Ceder, G.; Persson, K. A. Commentary: The Materials Project: A materials genome approach to accelerating materials innovation. *APL Materials* **2013**, *1*, 011002.

3 . Kingsbury, R. S.; Rosen, A. S.; Gupta, A. S.; Munro, J. M.; Ong, S. P.; Jain, A.; Dwaraknath, S.; Horton, M. K.; Persson, K. A. A flexible and scalable scheme for mixing computed formation energies from different levels of theory. *npj Computational Materials* **2022**, *8*, 195.

4 . Saßnick, H.-D.; Cocchi, C. Exploring cesium–tellurium phase space via high-throughput calculations beyond semi-local density-functional theory. *The Journal of Chemical Physics* **2022**, *156*, 104108.

5 . Aykol, M.; Dwaraknath, S. S.; Sun, W.; Persson, K. A. Thermodynamic limit for synthesis of metastable inorganic materials. *Science Advances* **2018**, *4*, eaaq0148.

6 . Li, Q.; Fu, N.; Omee, S. S.; Hu, J. MD-HIT: Machine learning for material property prediction with dataset redundancy control. *npj Computational Materials* **2024**, *10*, 245.

7 . Witman, M. D.; Schindler, P. MatFold: systematic insights into materials discovery models' performance through standardized cross-validation protocols. *Digital Discovery* **2025**, *4*, 625-635.

8 . Shimakawa, H.; Kumada, A.; Sato, M. Prevention of Leakage in Machine Learning Prediction for Polymer Composite Properties. *Journal of Chemical Information and Modeling* **2024**, *64*, 3621-3629.

9 . Muckley, E. S.; Saal, J. E.; Meredig, B.; Roper, C. S.; Martin, J. H. Interpretable models for extrapolation in scientific machine learning. *Digital Discovery* **2023**, *2*, 1425-1435.

10 . Bender, A.; Schneider, N.; Segler, M.; Patrick Walters, W.; Engkvist, O.; Rodrigues, T. Evaluation guidelines for machine learning tools in the chemical sciences. *Nature Reviews Chemistry* **2022**, *6*, 428-442.

11 . Xu, Y.; Liaw, A.; Sheridan, R. P.; Svetnik, V. Development and Evaluation of Conformal Prediction Methods for Quantitative Structure–Activity Relationship. *ACS Omega* **2024**, *9*,

29478-29490.
12 . Moscovich, A.; Rosset, S. On the Cross-Validation Bias due to Unsupervised Preprocessing. *Journal of the Royal Statistical Society Series B: Statistical Methodology* **2022**, *84*, 1474-1502.
13 . Geurts, P.; Ernst, D.; Wehenkel, L. Extremely randomized trees. *Machine Learning* **2006**, *63*, 3-42.
14 . Saito, T.; Rehmsmeier, M. The Precision-Recall Plot Is More Informative than the ROC Plot When Evaluating Binary Classifiers on Imbalanced Datasets. *PLOS ONE* **2015**, *10*, e0118432.
15 . Au, Q.; Herbinger, J.; Stachl, C.; Bischl, B.; Casalicchio, G. Grouped feature importance and combined features effect plot. *Data Mining and Knowledge Discovery* **2022**, *36*, 1401-1450.
16 . Wang, S.; Gong, S.; Böger, T.; Newnham, J. A.; Vivona, D.; Sokseiha, M.; Gordiz, K.; Aggarwal, A.; Zhu, T.; Zeier, W. G.; Grossman, J. C.; Shao-Horn, Y. Multimodal Machine Learning for Materials Science: Discovery of Novel Li-Ion Solid Electrolytes. *Chemistry of Materials* **2024**, *36*, 11541-11550.
17 . Tsamardinos, I.; Greasidou, E.; Borboudakis, G. Bootstrapping the out-of-sample predictions for efficient and accurate cross-validation. *Machine Learning* **2018**, *107*, 1895-1922.
18 . He, J.; Yue, T.; Kim, Y. L.; Li, Y. High-throughput screening of piezoelectric hybrid organic–inorganic perovskites via density-functional theory and machine learning. *Journal of Materials Chemistry A* **2026**, *14*, 24610-24623.
19 . Kavalsky, L.; Hegde, V. I.; Meredig, B.; Viswanathan, V. A multiobjective closed-loop approach towards autonomous discovery of electrocatalysts for nitrogen reduction. *Digital Discovery* **2024**, *3*, 999-1010.
20 . Harris, C. R.; Millman, K. J.; van der Walt, S. J.; Gommers, R.; Virtanen, P.; Cournapeau, D.; Wieser, E.; Taylor, J.; Berg, S.; Smith, N. J.; Kern, R.; Picus, M.; Hoyer, S.; van Kerkwijk, M. H.; Brett, M.; Haldane, A.; del Río, J. F.; Wiebe, M.; Peterson, P.; Gérard-Marchant, P.; Sheppard, K.; Reddy, T.; Weckesser, W.; Abbasi, H.; Gohlke, C.; Oliphant, T. E. Array programming with NumPy. *Nature* **2020**, *585*, 357-362.

21 . P. Giannozzi, S. Baroni, N. Bonini, M. Calandra, R. Car, C. Cavazzoni, D.Ceresoli, G. L. Chiarotti, M. Cococcioni, I. Dabo, A. Dal Corso, S. de Gironcoli, S. Fabris, G. Fratesi, R. Gebauer, U. Gerstmann, C.Gougoussis, A. Kokalj, M. Lazzeri, L. Martin-Samos, N. Marzari, F. Mauri, R. Mazzarello, S. Paolini, A. Pasquarello, L. Paulatto, C. Sbraccia, S. Scandolo, G. Sclauzero, A. P. Seitsonen, A. Smogunov, P. Umari, and R. M. Wentzcovitch. QUANTUM ESPRESSO: A modular and opensource software project for quantum simulations of materials, *J. Phys.Condens. Matter.* ***2009,***21, 395502.
